# Characterization of the Mechanisms of Daptomycin Resistance among Gram-Positive Bacterial Pathogens by Multidimensional Lipidomics

**DOI:** 10.1128/mSphere.00492-17

**Published:** 2017-12-13

**Authors:** Kelly M. Hines, Adam Waalkes, Kelsi Penewit, Elizabeth A. Holmes, Stephen J. Salipante, Brian J. Werth, Libin Xu

**Affiliations:** a Department of Medicinal Chemistry, University of Washington, Seattle, Washington, USA; b Department of Laboratory Medicine, University of Washington, Seattle, Washington, USA; c Department of Pharmacy, University of Washington, Seattle, Washington, USA; U.S. Centers for Disease Control and Prevention

**Keywords:** PgsA, antibiotic resistance, daptomycin, Gram-positive bacteria, ion mobility-mass spectrometry, lipidomics, whole-genome sequencing

## Abstract

Previous work suggests that altered lipid metabolism may be associated with daptomycin resistance in Gram-positive pathogens, but lipidomic changes underlying resistance are not fully understood. We performed untargeted lipidomics by using three-dimensional hydrophilic interaction liquid chromatography-ion mobility-mass spectrometry (HILIC-IM-MS) to characterize alterations in the lipidomes of daptomycin-susceptible and -resistant isogenic strain pairs of *Enterococcus faecalis*, *Staphylococcus aureus*, and *Corynebacterium striatum*. We first validated the HILIC-IM-MS method by replicating the expected alterations of phospholipid metabolism in the previously studied *E. faecalis* strain pairs, such as reduced phosphatidylglycerols (PGs), while also revealing additional changes in cardiolipins (CLs), lysyl-PGs, and glycolipids. Whole-genome sequencing of the *S. aureus* and *C. striatum* strains found that daptomycin resistance was associated with mutations in *pgsA*, which encodes phosphatidylglycerophosphate synthase, as well as mutations in genes affecting fatty acid biosynthesis and cell wall metabolism. Lipidomics revealed significantly decreased levels of PGs, CLs, and amino acid-modified PGs, as well as accumulation of lipids upstream of PGs, such as glycolipids and phosphatidic acids, in the resistant strains. Notably, the glycolipids, diglucosyldiacylglycerols, were significantly elevated in a fatty acid-dependent manner in the daptomycin-resistant *S. aureus* strain. In daptomycin-resistant *C. striatum*, which has a unique cell envelope architecture, the glycolipids, glucuronosyldiacylglycerols, and phosphatidylinositols were significantly elevated. These results demonstrate that alteration of lipid metabolism via mutations in *pgsA* is a common mechanism of daptomycin resistance in two distinct species of Gram-positive bacteria and point to the potential contribution of altered glycolipid and fatty acid compositions to daptomycin resistance.

**IMPORTANCE** This work comprehensively characterizes lipidomic changes underlying daptomycin resistance in three Gram-positive bacterial species, *E. faecalis*, *S. aureus*, and *C. striatum*, by using a novel three-dimensional lipidomics methodology based on advanced mass spectrometry. We demonstrated a number of advantages of our method in comparison with other methods commonly used in the field, such as high molecular specificity, sensitivity, and throughput. Whole-genome sequencing of the *S. aureus* and *C. striatum* strains identified mutations in *pgsA*, which encodes phosphatidylglycerophosphate synthase, in both resistant strains. Lipidomics revealed significantly decreased levels of lipids downstream of PgsA, as well as accumulation of lipids upstream of PgsA in the resistant strains. Furthermore, we found that changes in individual molecular species of each lipid class depend on the their specific fatty acid compositions. The characteristic changes in individual lipid species could be used as biomarkers for identifying underlying resistance mechanisms and for evaluating potential therapies.

## Abstract (precis)

This work comprehensively characterizes lipidomic changes underlying daptomycin resistance in three Gram-positive bacterial species, *E. faecalis*, *S. aureus*, and *C. striatum*, by using a novel three-dimensional lipidomics methodology based on advanced mass spectrometry. We demonstrated a number of advantages of our method in comparison with other methods commonly used in the field, such as high molecular specificity, sensitivity, and throughput. Whole-genome sequencing of the *S. aureus* and *C. striatum* strains identified mutations in *pgsA*, which encodes phosphatidylglycerophosphate synthase, in both resistant strains. Lipidomics revealed significantly decreased levels of lipids downstream of PgsA, as well as accumulation of lipids upstream of PgsA in the resistant strains. Furthermore, we found that changes in individual molecular species of each lipid class depend on the their specific fatty acid compositions. The characteristic changes in individual lipid species could be used as biomarkers for identifying underlying resistance mechanisms and for evaluating potential therapies.

## INTRODUCTION

Antibiotic resistance is a major threat to public health, affecting over 2 million Americans and resulting in over 23,000 deaths per year (1). More than 11,000 of these deaths are attributed to methicillin-resistant *Staphylococcus aureus* (MRSA) alone (1). The glycopeptide vancomycin continues to be the drug of choice for treating invasive MRSA infections, but vancomycin-nonsusceptible phenotypes have emerged, including vancomycin-intermediate *S. aureus* (VISA) and heterogeneous VISA (2). Vancomycin resistance has also become prevalent among enterococci, and it is now estimated that 30% of all enterococcal infections in the United States are caused by vancomycin-resistant enterococci (1).

Daptomycin is a novel antimicrobial with bactericidal activity against most Gram-positive bacteria that plays an important role in the treatment of serious infections caused by vancomycin-nonsusceptible pathogens (3, 4). Daptomycin is an amphiphilic lipopeptide that consists of a cyclic polypeptide with 13 amino acids and a decanoyl fatty acid (FA) tail (5, 6). Its mechanism of action has been postulated to be direct interaction with the cell membrane that leads to membrane distortion and an indirect impact on cell wall synthesis by changing the localization of related proteins, all of which lead to ultimate depolarization of the cell membrane and cell death (6–11). The formation of a complex with calcium is necessary for the activity of daptomycin, which promotes the formation of micelles to deliver daptomycin to the cell membrane and facilitate its insertion (7, 8, 12–14). Oligomerization of the daptomycin-calcium complexes in the cell membrane is a critical step in the sequence of actions and is dependent on the presence of the phospholipids phosphatidylglycerols (PGs), a class of negatively charged lipids (7–9).

However, daptomycin resistance (although daptomycin “nonsusceptibility” is the technically accurate term, “resistance” was used for ease of presentation) has emerged in a variety of clinically relevant species of Gram-positive pathogens (15, 16), which leaves clinicians limited options for treatment. Various gene mutations, mostly involved in either cell wall or lipid synthesis, have been observed among different daptomycin-resistant species (5, 6). In *S. aureus*, gain-of-function mutations in *mprF* are commonly observed, resulting in increased levels of total lysyl-PGs (17–21). Mutations in genes involved in lipid synthesis, such as *cls* (cardiolipin [CL] synthases) and *pgsA* (PG synthase) (22), and two-component regulatory systems of cell envelope homeostasis, *vraSR* and *yycFG* (23, 24), have also been shown to contribute to daptomycin resistance. Upregulation of the *tagA* gene (25, 26), which is involved in teichoic acid synthesis, and the *dlt* operon (20, 27, 28), which controls d-alanylation of teichoic acid and LTA, has also been linked with daptomycin resistance. The physiological changes resulting from the alteration of one or more of these genes are consistent with an increased positive charge in the cell envelope, which reduces binding of the cationic daptomycin-calcium complex (5, 6). Separately, mutations in genes involved in lipid metabolism and cell envelope regulation have also been implicated in daptomycin resistance in other Gram-positive organisms, such as *Bacillus subtilis* (*liaFSR* and *pgsA*) (29), *Enterococcus faecalis* (*liaFSR*, *cls*, and *gdpD*) (15), *Enterococcus faecium* (*liaFSR*, *cls*, and *yycFG*) (30), and *Streptococcus mitis/oralis* (*cdsA*) (31, 32). The common metabolic feature associated with this category of mutation is decreased levels of PGs, but changes in lysyl-PGs or CLs have not been consistently observed (17, 31–34).

Comprehensive comparison of the lipidomic signatures differentiating susceptible and resistant isogenic strains and among different species could provide a metabolic indicator of both common and uncommon mechanisms of resistance to daptomycin but has not yet been performed. Analysis of bacterial lipids has typically been carried out by two-dimensional (2D) thin-layer chromatography (TLC) (17, 34, 35), but this method is labor intensive, lacks molecular specificity, and requires large amounts of analytical materials. 2D TLC normally quantifies each lipid class as a whole instead of individual molecular species and does not allow quantification of lipid classes without a phosphate group because a colorimetric method for phosphate is typically used for quantification (17, 34, 35). Untargeted liquid chromatography-mass spectrometry (LC-MS) has also been applied to the analysis of bacterial lipids with improved sensitivity and specificity (36), but it remains difficult to resolve overlapping and similar-mass lipid species. More recently, Rashid et al. used a combination of targeted and untargeted LC-MS methods for comprehensive lipidomic studies of *E. faecalis*, but the workflow is time-consuming as separate sample preparations and LC-MS methods are needed for glycerolipids, phospholipids, and CLs (37). To address the limitations of the previous methods, we recently developed a 3D lipidomics approach based on hydrophilic interaction LC-ion mobility-MS (HILIC-IM-MS) that enables high-throughput and high-specificity lipid analysis (38). HILIC separates lipid classes on the basis of their head group polarity (on a scale of seconds), and IM separates lipid ions on the basis of their gas phase structures or collision cross sections (CCSs) as they pass through an inert gas background (on a scale of milliseconds). Importantly, CCS values can be used as an additional physical property for lipid identification (38, 39). However, this method has not been applied to the characterization of bacterial lipidomes.

Here, we collected or generated three isogenic pairs of daptomycin-susceptible and -resistant species: a clinically derived *E. faecalis* pair with an MIC difference of 12-fold (15), an *in vitro*-derived MRSA strain pair with an MIC difference of 64-fold, and a clinically derived *Corynebacterium striatum* pair with an astonishing MIC difference of >2,000-fold (40). We first evaluated the previously studied *E. faecalis* strain pair (S613 [daptomycin MIC, 2 µg/ml] and R712 [daptomycin MIC, 16 µg/ml]) to assess the ability of the HILIC-IM MS method to replicate the previously published lipidomic findings on these strains and compile a collection of CCS values of lipids commonly observed in Gram-positive bacteria for confident identification of these species. Whole-genome sequencing of the MRSA and *C. striatum* strain pairs was performed, revealing mutations in genes associated with phospholipid metabolism, FA synthesis, and cell wall metabolism in the resistant strains. We then characterized the lipidomic changes associated with daptomycin resistance in the MRSA and *C. striatum* strain pairs, which revealed that several lipidomic alterations were conserved across these distinct species with daptomycin resistance and that these changes are consistent with the genetic mutations underlying their resistance.

## RESULTS

### HILIC-IM-MS for untargeted lipidomics.

**FIG 1:**
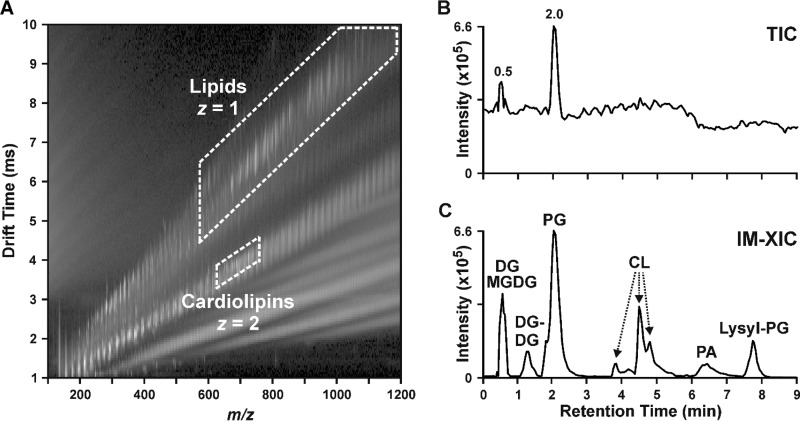
Lipidomics analysis by HILIC-IM-MS. (A) 2D plot of IM drift time versus *m/z* shows that singly charged (*z* = 1) lipids occupy a region distinct from that of doubly charge (*z* = 2) lipids such as CLs. (B) The TIC (0 to 9 min of a 12-min run) from the negative-mode analysis of a lipid mixture suffers from high baseline noise. (C) Extraction of the regions containing *z* = 1 and *z* = 2 lipids (dashed outlines) generated an IM-XIC containing only the lipid signals from the mixture of DGs, MGDGs, DGDGs, PGs, CLs, PAs, and lysyl-PGs.

We have recently demonstrated the utility of HILIC-IM-MS for untargeted lipidomics analyses of Neuro2a neuroblastoma cells exposed to environmental chemicals in which the major classes of mammalian lipids are separated by polarity in the chromatographic dimension and by structure in the IM dimension (38). On the basis of our promising mammalian lipidomics analysis results, we believe that the HILIC-IM-MS method offers several advantages to the studies of bacterial lipidomics, with minor modifications. Figure 1 demonstrates the HILIC-IM-MS method for a mixture of lipids similar to those observed in Gram-positive bacteria, including diacylglycerols (DGs), monogalactosyldiacylglycerols (MGDGs), digalactosyldiacylglycerols (DGDGs), PGs, cardiolipins (CLs), phosphatidic acids (PAs), and lysyl-PGs. In the IM-MS dimension (Fig. 1A), lipid species are first separated on the basis of charge state, where doubly charged (*z* = 2) lipids such as CLs have shorter drift times than singly charged (*z* = 1) lipids with similar *m/z* ratios, such as PGs. Both regions highlighted in Fig. 1A can be extracted to yield an IM-extracted ion chromatogram (IM-XIC) that contains only lipid species, as shown in Fig. 1C. This has the effect of reducing the high background noise in the total ion chromatogram (TIC) shown in Fig. 1B, where only the chromatographic peaks of the most intense lipid species (i.e., PGs) can be visibly discerned. CCSs from the IM dimension may serve as additional validating data for the tentative identification of lipid species. We have compiled the CCS values (see Tables S1 and S2 in the supplemental material) for the lipid species shown in Fig. 1 and the lipids observed in *E. faecalis*, *S. aureus*, and *C. striatum*, many of which are reported here for the first time (87 in positive mode, 73 in negative mode). Collectively, this rapid multidimensional separation strategy for lipidomics provides an improved signal-to-noise ratio, greater specificity for lipids, and detailed molecular information for lipid identification.

10.1128/mSphere.00492-17.5 TABLE S1  CCS values from the analysis of the lipid standard mixture in negative and positive modes. Superscript letter a: interday average CCS, *n* = 4. Download TABLE S1, PDF file, 0.1 MB. Copyright © 2017 Hines et al. 2017 Hines et al. This content is distributed under the terms of the Creative Commons Attribution 4.0 International license.

10.1128/mSphere.00492-17.6 TABLE S2  CCS values for lipids identified in *E. faecalis*, *S. aureus* and *C. striatum* strain pairs. Superscript letters: a, intraday average CCS, *n* ≥3; b, K. M. Hines, J. M. Herron, and L. Xu, J Lipid Res 58:809–819, 2017, https://doi.org/10.1194/jlr.D074724; c, see Table S1. Download TABLE S2, PDF file, 0.3 MB. Copyright © 2017 Hines et al. 2017 Hines et al. This content is distributed under the terms of the Creative Commons Attribution 4.0 International license.

To adapt the HILIC-IM-MS method from mammalian to bacterial systems, we first evaluated a reconstructed mixture of commercial lipid extracts from various species that contained one major CL species containing only 18:2 FAs (CL 72:8) extracted from heart tissue. This reconstructed lipid mixture was handled similarly to a prepared bacterial lipid extract prior to HILIC-IM-MS analysis, where a small amount of the mixture in chloroform was transferred to an LC vial, dried under argon, and reconstituted in HILIC mobile phase A. The results for these standards were as expected, with a sensitivity similar to that of our analysis of mammalian lipids (38). However, when we first analyzed the lipid extracts of Gram-positive bacteria, the anticipated CL species were missing. The analysis of a commercial *Escherichia coli* CL extract produced a similar result, where very little CL abundance was observed. We determined that the FA composition of CLs in bacteria was significantly more saturated and tended to be shorter than that of mammalian CLs, and thus, the HILIC A mobile phase (i.e., 95% acetonitrile and 5% water with 5 mM ammonium acetate) was not solubilizing the bacterial CLs as well as CL 72:8 from heart tissue. By modifying the reconstitution solvent of the bacterial lipids to a mixture of 60% methanol, 20% acetonitrile, and 20% 1 mM ammonium acetate as used in previous studies (41, 42), we were able to observe CLs in the commercial *E. coli* extract and extracts of *S. aureus* N315 under the same gradient conditions. However, the injection of a large amount of polar methanol into the high-acetonitrile initial conditions of the HILIC gradient resulted in some peak broadening for the early-eluting lipid species, such as PGs. After further optimization, we found that a 2:1 mixture of acetonitrile and methanol yielded similar CL abundances and resulted in only a minor broadening of chromatographic peaks. This modification in the sample preparation enabled all of the relevant bacterial lipid species to be evaluated in a single run.

### Daptomycin-resistant *E. faecalis*.

**FIG 2:**
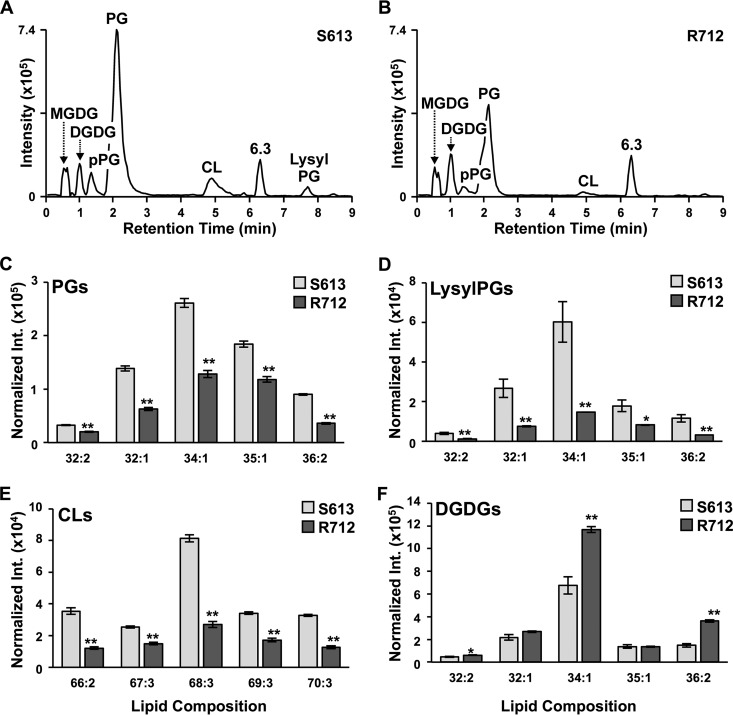
Results of the analysis of *E. faecalis* strains S613 and R712 by the HILIC-IM-MS lipidomics method. Negative-mode IM-XICs for daptomycin-susceptible *E. faecalis* strain S613 (raw intensity of 82-mg dry pellet shown) (A) and daptomycin-resistant *E. faecalis* strain R712 (raw intensity of 69-mg dry pellet shown) (B) reveals that the predominant lipid species in *E. faecalis* are PGs, CLs, DGDGs, MGDGs, and lysyl-PGs. PG (C), lysyl-PG (D), and CL (E) species are reduced in R712 regardless of the FA composition. (F) Individual DGDG species are reduced in R712 in a manner that is dependent upon the FA composition. Data shown in panels C to F are normalized to dry pellet weight. The FA compositions of individual lipid species were determined (total carbon number and unsaturation degree of the FAs in each lipid are shown; detailed FA compositions can be found in supplemental material), and the statistical significance of differences in intensity (Int.) was determined by Student's *t* test. *, *P* ≤ 0.05; **, *P* ≤ 0.005.

Well-characterized *E. faecalis* strains S613 and R712 were selected for lipidomic evaluation and validation of our HILIC-IM-MS approach since these strains have been previously evaluated with respect to their lipid changes and subjected to whole-genome sequencing (15, 34). The results of the HILIC-IM-MS analysis of the *E. faecalis* strain pair are shown in Fig. 2. While the same classes of lipid species were observed in both S613 (Fig. 2A) and R712 (Fig. 2B), the relative abundances of several lipid species were significantly altered in daptomycin-resistant strain R712. As expected with daptomycin resistance, the most notable difference was a significant reduction of PGs (Fig. 2C, 1.4- to 5-fold reduction) in R712. In addition, we observed similar fold reductions in plasmalogen PGs (i.e., ether-linked PGs, pPGs), lysyl-PGs (Fig. 2D), and CLs (Fig. 2E). The decrease in PGs is consistent with previous work by Mishra et al. with the same strains (34), but they did not analyze individual PG species and lipid classes of DGDGs and MGDGs and did not observe significant changes in lysyl-PGs and CLs. Rashid et al. observed that both PGs and lysl-PGs were decreased in *E. faecalis* strain OG1RF with daptomycin resistance relative to a nonresistant matching strain, but changes in other classes of lipids were not reported (37). In this study, DGDGs, the glycolipid anchors of lipoteichoic acid (LTA), and precursor MGDGs were found to be elevated in R712 (Fig. 2F; Table S3, 1.2- to 2.4-fold increases), which is also consistent with the increase in glycerophospho-DGDGs (GP-DGDGs) reported by Mishra et al. (34). The individual lipid species with the highest abundance was that with an FA composition of 34:1 (total carbon number and unsaturation degree of the FAs in each lipid with two FAs) across all lipid classes (for CL, 68:3). Targeted MS/MS experiments determined the specific FAs in the 34:1 lipids to be a combination of 18:1 and 16:0, with some 16:1 FAs present for the most abundant CL, CL 68:3 (FA compositions of other species in Fig. 2 are shown in Table S3). The DGDGs elevated in R712 were all found to contain at least one 18:1 FA (DGDG 32:2, 32:1 [not significant], 34:1, and 36:2).

10.1128/mSphere.00492-17.7 TABLE S3  Individual lipid species altered in *E. faecalis* strains S613 (daptomycin susceptible) and R712 (daptomycin resistant). Superscript letters: a, FAs determined from targeted MS/MS experiments in negative ionization mode; b, significance determined by Student's *t* test, *P* ≤ 0.05. Download TABLE S3, PDF file, 0.1 MB. Copyright © 2017 Hines et al. 2017 Hines et al. This content is distributed under the terms of the Creative Commons Attribution 4.0 International license.

### Daptomycin resistance in MRSA.

Because of the lack of a clinical isogenic pair of MRSA strains with only daptomycin susceptibility changes in our strain collection, we used the *in vitro* serial passage method to select a daptomycin-nonsusceptible mutant of well-characterized laboratory strain N315 similar to previous studies (43). A stable derivative of MRSA strain N315 with a 64-fold reduction in daptomycin susceptibility (N315-D8; MIC, 8 µg/ml) was isolated and characterized. Whole-genome sequencing was performed to determine the underlying genetic mutations contributing to the daptomycin resistance observed in N315-D8. Single nucleotide polymorphisms were identified in seven predicted gene products of N315-D8 (Table 1). Notably, two genes involved in phospholipid biosynthesis, *pgsA* and *mprF*, were found to be mutated in N315-D8. CDP-DG-glycerol-3-phosphate 3-phosphatidyltransferase A (PgsA) catalyzes the transfer of glycerol-3-phosphate to CDP-DG to form phosphatidyl glycerophosphate (PGP). Multiprotein resistance factor (MprF) performs lysinylation of PGs to form lysyl-PGs and is also responsible for the flipping of lysyl-PGs from the inner to the outer membrane leaflet. Furthermore, YycG (also known as WalK or VicK) is a histidine kinase in the YycFG (or WalRK or VicRK) two-component system that regulates cell wall metabolism and FA biosynthesis (44–47). Thus, one would expect significant changes in the lipid metabolism of the N315-D8 strain.

**TABLE 1:** Genetic mutations identified in *in vitro*-derived *S. aureus* strain N315 with daptomycin resistance (N315-D8) relative to the parent N315 strain

Predicted gene product	Nucleotide change in N315-D8	Predicted amino acid change in N315-D8^a^	Predicted protein function(s)
YycG	G → A (1278)	M426I	FA biosynthesis, cell wall biosynthesis
PgsA	A → G (403)	K135E	PG biosynthesis
MprF	C → T (2476)	L826F	Lysyl-PG biosynthesis
SA0567	C → T (865)	Q289*	Iron complex transport
NorA	G → C (737)	G246A	Quinolone resistance
Rnr	G → T (1819)	E607*	RNase
SpoIIIE	C2059 deletion	Q687 frameshift	DNA translocase

a Asterisks indicate a change to a stop codon.

**FIG 3:**
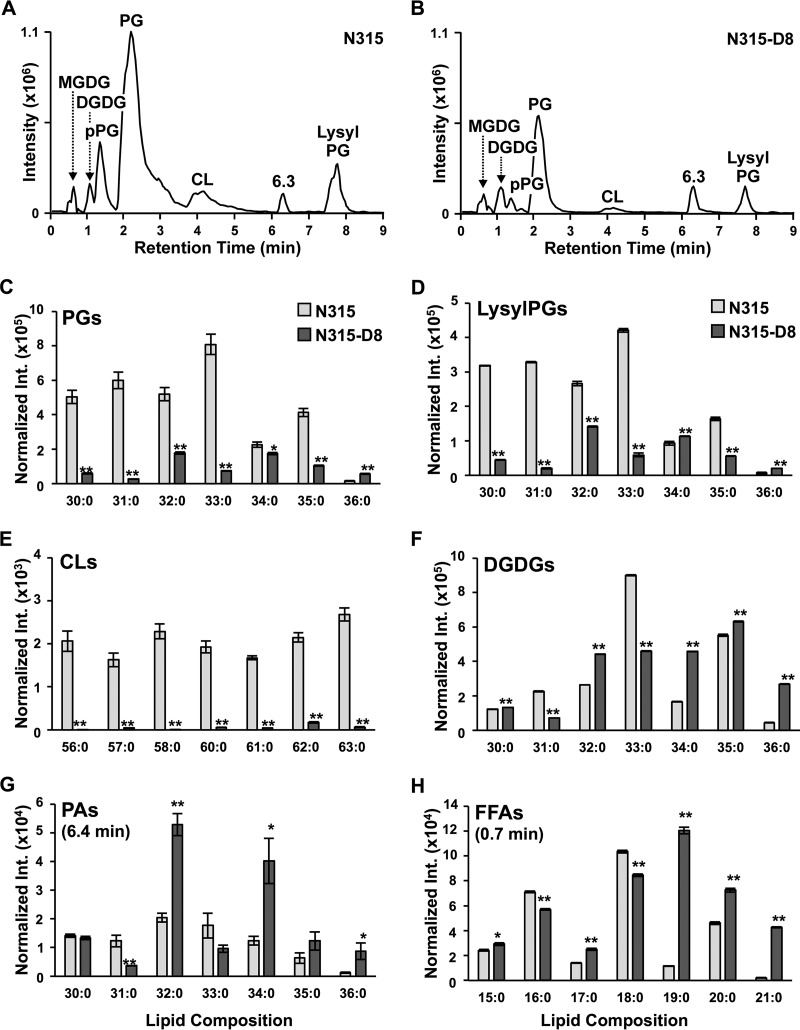
Results of analyses of daptomycin-susceptible (N315) and daptomycin-resistant (N315-D8) *S. aureus* by HILIC-IM-MS. Negative-mode IM-XICs of N315 (raw intensity [Int.] of 101-mg dry pellet shown) (A) and N315-D8 (raw intensity of 77-mg dry pellet shown) (B) reveal that PGs and lysyl-PGs are the major lipid species in *S. aureus* N315. The abundances of PGs 30:0 to 36:0 (C), lysyl-PGs 30:0 to 36:0 (D), CLs 56:0 to 63:0 (excluding 59:0) (E), PAs 30:0 to 36:0 in N315 and N315-D8 (F), and DGDGs 30:0 to 36:0 (G) are shown. (H) The abundances of FFAs that were differentially abundant in N315 and N315-D8 are also shown. Data shown in panels C to H are normalized to dry pellet weight, and error bars represent the standard deviation of the mean. The significance of differences was determined by Student's *t* test. *, *P* ≤ 0.05; **, *P* ≤ 0.005.

**FIG 4:**
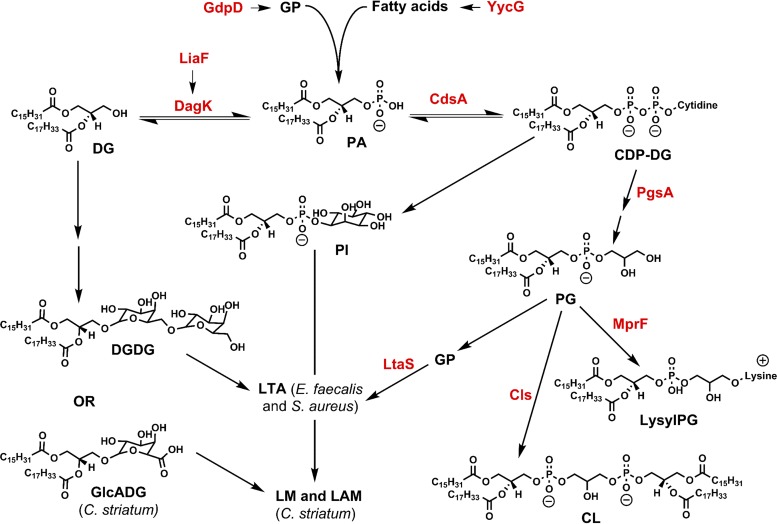
Selected pathways of phospholipid and glycerolipid metabolism. PgsA, CDP-DG–glycerol-3-phosphate 3-phosphatidyltransferase; DagK, DG kinase; GP, *sn*-glycerol-3-phosphate. Mutations in *gdpD* and *liaF* (15), *pgsA*, *yycG* and *mprF* (this work), and *pgsA* (this work) were found in daptomycin-resistant *E. faecalis* (R712), *S. aureus* (N315-D8), and *C. striatum* (W49297), respectively, in comparison with the matching daptomycin-susceptible strains.

Lipidomic analysis was carried out to compare the N315-D8 strain with the parent N315 strain (N315; MIC, 0.125 µg/ml) by the HILIC-IM-MS method to assess the metabolic consequences of the genetic mutations associated with these susceptibility changes. As shown in Fig. 3A and B, the lipid composition of N315 consisted of PGs, lysyl-PGs, pPGs, CLs, DGDGs, and MGDGs. The levels of PGs were significantly reduced overall in N315-D8 relative to those in the parent N315 strain (Fig. 3C). However, the magnitude of reduction varied on the basis of the FA composition of the PG species. For example, the most abundant PG in N315, PG 33:0, decreased by 10-fold (*t* test *P* = 6.2 × 10^−5^, Table S4) in N315-D8. PGs 32:0, 34:0, and 35:0 (FA composition shown in Table S4) also decreased in N315-D8 (by 1.25- to 3-fold) but to a lesser extent than PG 33:0. In contrast, the level of a minor component of PGs, PG 36:0, was significantly higher in N315-D8 (3.6-fold, *t* test *P* = 2.3 × 10^−5^) than in N315. The levels of lysyl-PGs (Fig. 3D) and CLs (Fig. 3E), which are both derived from PGs (Fig. 4), were lower overall in N315-D8 than in the parent N315. A similar pattern of abundances was observed among individual lysyl-PG species, where lysyl-PG 36:0 was elevated in N315-D8 (3.1-fold, *t* test *P* = 2.3 × 10^−6^) to a similar degree as PG 36:0. The level of lysyl-PG 34:0 was also elevated in N315-D8 (1.2-fold, *t* test *P* = 4.0 × 10^−4^), whereas those of other lysyl-PG species were reduced to various degrees (2- to 25-fold).

10.1128/mSphere.00492-17.8 TABLE S4  Individual lipid species altered in *S. aureus* strains N315 (daptomycin susceptible) and N315-D8 (daptomycin resistant). Superscript letters: a, FAs determined from targeted MS/MS experiments in negative ionization mode; b, significance determined by Student's *t* test, *P* ≤ 0.05. Download TABLE S4, PDF file, 0.2 MB. Copyright © 2017 Hines et al. 2017 Hines et al. This content is distributed under the terms of the Creative Commons Attribution 4.0 International license.

FA-dependent intensity profiles were also observed in several other classes of lipid species. The levels of a majority (64%) of the DGDG species observed were elevated in N315-D8 (Fig. 3F). Among the seven most abundant DGDGs, the largest fold increases were observed in DGDGs 32:0, 34:0, and 36:0 (1.7- to 6.1-fold increases, *t*-test *P* values in Table S4). The major DGDG species that were decreased in N315-D8 were DGDG 33:0, the most abundant DGDG in parent N315, and DGDG 31:0 (2- to 3-fold, *t*-test *P* values in Table S4). MGDGs were observed at much lower levels but were altered similarly to those of matching DGDGs. Furthermore, PAs were also affected in a manner similar to that of DGDGs in N315-D8 (Fig. 3G), with elevated (2- to 7-fold) levels of PAs 32:0, 34:0, 35:0 (not significant), and 36:0 and decreased (2- to 3-fold) levels of PAs 31:0 and 33:0 (not significant).

Given the apparent FA-dependent abundance profiles of phospholipid and glycolipids species in N315-D8, targeted HILIC-IM-tandem MS (MS/MS) experiments were performed to determine the FA compositions of individual lipid species. FA 15:0 was found to be the most prevalent FA in all lipid classes, while the second FA in diacyl phospholipids and glycolipids varied from FA 15:0 to FA 21:0 in those species shown in Fig. 3C to G. For example, the PA and DGDG species with FA compositions of 34:0, 35:0, and 36:0, all found to be elevated in N315-D8, were composed of FAs 15:0, 19:0, 20:0, and 21:0 (Table S4). A number of free FAs (FFAs) were identified by the HILIC-IM-MS method by comparing their retention times with those of the authentic standards, and the intensities of selected FAs are shown in Fig. 3H (additional FFAs are shown in Table S4). While the FFA 16:0 and 18:0 abundances were decreased in N315-D8, the abundances of 19:0, 20:0, and 21:0 were significantly elevated in N315-D8 by 10-, 1.6-, and 20-fold, respectively.

### Daptomycin-resistant *C. striatum*.

The previously described clinically derived strain pairs of daptomycin-susceptible (W40308; MIC, 0.125 µg/ml) and resistant (W49297; MIC, >256 µg/ml) *C. striatum* were used for this study (40). Whole-genome sequencing was performed to assess the mutations associated with daptomycin resistance in *C. striatum*. Six predicted protein mutations were observed in W49297 (Table 2) relative to the genome of W40308. Significantly, a frameshift mutation in *pgsA2*, which encodes a putative CDP-diacylglycerol-glycerol-3-phosphate 3-phosphatidyltransferase 2, was observed. Other mutations in W49297 indicate modifications of the central carbon (*gabD1*), folate (*adgB*), and ascorbate (*dkgA*) metabolism, as well as NAD biosynthesis (*pncB1*) and FA β-oxidation (*pcaF*) in daptomycin-resistant *C. striatum*.

**TABLE 2:** Genetic mutations identified in daptomycin-resistant *C. striatum* strain W49297 in comparison with susceptible strain W40308

Predicted gene product	Nucleotide change in W49297	Predicted amino acid change in W49297^a^	Predicted protein function(s)
PgsA2	T520 deletion	F174 frameshift	PG biosynthesis
AdgB	C → T (784)	R262C	Folate catabolism
DkgA	T → G (609)	D206E	Ascorbate biosynthesis
GabD1	G → T (1001)	G334V	Tricarboxylic acid cycle
PncB1	GCAAGCAGCTCGACGA deletion (845–860)	K283*	NAD biosynthesis
PcaF	G → A (173)	G57D	3-Ketoacyl coenzyme A thiolase, FA β-oxidation

a Asterisk indicates a change to a stop codon.

**FIG 5:**
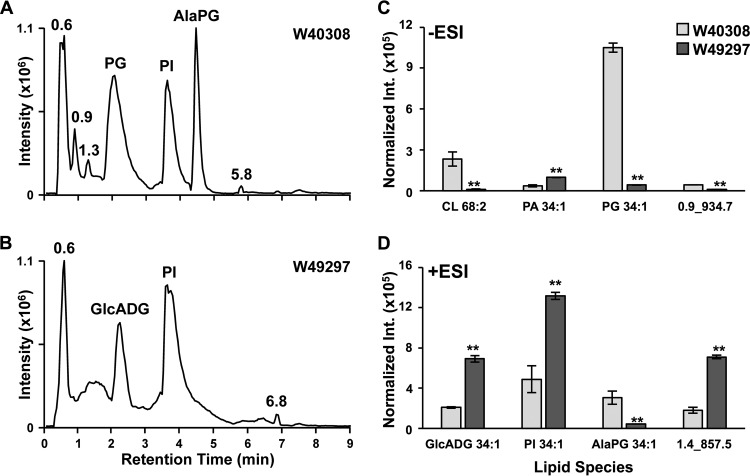
Results of analyses of daptomycin-susceptible (W40308) and -resistant (W49297) *C. striatum* by HILIC-IM-MS. (A) Positive-mode IM-XIC of W40308 (raw intensity [Int.] of 183-mg dry pellet shown) reveals that AlaPGs, PGs, and PIs are the major lipid species in daptomycin-susceptible *C. striatum*. (B) The major lipid species in daptomycin-resistant *C. striatum* strain W49297 (raw intensity of 41-mg dry pellet shown) are GlcADGs and PIs in positive mode. (C) The abundances of the major lipid species in *C. striatum* observed in the negative-mode (−ESI) HILIC-IM-MS analysis. (D) The abundances of the major lipid species observed in the positive-mode (+ESI) HILIC-IM-MS analysis. Data shown in panel C and D are normalized to dry pellet weight, and error bars represent the standard deviation of the mean. Significance was determined by Student's *t* test. **, *P* ≤ 0.005.

Lipidomics studies again revealed significant perturbation of lipid metabolism in the daptomycin-resistant strain relative to the susceptible *C. striatum* strain (40). Unlike the diverse FA compositions in *E. faecalis* and *S. aureus*, the major lipid species observed in *C. striatum* were PG 16:0/18:1 (*sn1* and *sn2* positions not determined), alanyl-PG 16:0/18:1 (AlaPG, see Fig. S1 for MS/MS spectra), and PI 16:0/18:1. The abundance of PG 16:0/18:1 (Fig. 5C, PG 34:1) in the resistant strain was 25-fold lower than that in the susceptible strain (Table S5, *t* test *P* = 2.1 × 10^−6^). AlaPG 16:0/18:1 and CLs 66:2 and 68:2 were similarly reduced in W49297 (Fig. 5C and D), as well as an unknown lipid species, 0.90_934.7*m/z*(−), that also had 16:0 and 18:1 FAs (see Fig. S2 for MS/MS spectra). The abundances of PI and PA species with FA compositions of 16:0/18:1 and 16:0/16:0 were increased in W49297 by 2- to 4-fold (*t*-test *P* values in Table S5). Two unknown lipid species were elevated to similar degrees in W49297, one of which was identified as glucuronosyl diacyglycerol (GlcADG) 16:0/18:1 (Fig. 5D) by MS/MS analysis (Fig. S3) (48). The identity of the second unknown lipid species, 1.36_857.58*m/z*(+) (Fig. 5D), could not be determined from the MS/MS analysis (Fig. S4).

10.1128/mSphere.00492-17.1 FIG S1  Targeted HILIC-IM-MS/MS experiments determined the identify of unknown lipid 4.46_820.57*m/z*(+) in *C. striatum* as AlaPG 16:0 to 18:1. (A) The fragments that confirmed the identity of 4.46_820.57*m/z*(+) are mapped onto the structure of AlaPG 16:0 to 18:1. (B) The targeted positive-mode MS/MS spectrum revealed *m/z* 146.09, 244.08, and 557.57 fragments that were characteristic of fragmentation of the AlaPG head group and phospholipid backbone, respectively, as shown in panel A. (C) The targeted negative-mode MS/MS spectrum revealed *m/z* 88.05, 255.25, and 281.27 fragments that were characteristic of the Ala modification and the FA composition, respectively, as shown in panel A. Download FIG S1, PDF file, 0.03 MB. Copyright © 2017 Hines et al. 2017 Hines et al. This content is distributed under the terms of the Creative Commons Attribution 4.0 International license.

10.1128/mSphere.00492-17.2 FIG S2  Targeted MS/MS spectra of the unknown lipid 0.90_934.84*m/z*(−) in *C. striatum* in the positive (A) and negative (B) modes. Both spectra suggest that the unknown lipid has a head group with a mass of 255 Da, and the negative-mode MS/MS spectrum confirms that the lipid contains FAs 16:0 and 18:1. Download FIG S2, PDF file, 0.03 MB. Copyright © 2017 Hines et al. 2017 Hines et al. This content is distributed under the terms of the Creative Commons Attribution 4.0 International license.

10.1128/mSphere.00492-17.3 FIG S3  Identification of GlcADG 34:1 by negative-mode IM-MS/MS fragmentation. (A) Structure of GlcADG 18:1/16:0 with characteristic negative-mode fragments in red. (B) IM-MS/MS spectrum of *m/z* 769 from *m/z* 150 to 200 to assess head group fragments. The *m/z* 175 fragment is a water loss from *m/z* 193 and corresponds to the GlcA head group. (C) IM-MS/MS spectrum of *m/z* 769 from *m/z* 210 to 310 to assess FA composition. (D) IM-MS/MS spectrum of *m/z* 769 from *m/z* 440 to 580 to assess fragments corresponding to the loss of one FA from the structure in panel A. Download FIG S3, PDF file, 0.04 MB. Copyright © 2017 Hines et al. 2017 Hines et al. This content is distributed under the terms of the Creative Commons Attribution 4.0 International license.

10.1128/mSphere.00492-17.4 FIG S4  Targeted MS/MS spectra of unknown lipid 1.36_857.58*m/z*(+) in *C. striatum* in the positive (A) and negative (B) modes. Both spectra suggest that the unknown lipid has a head group with a mass of 258 Da, where *m/z* 281 in the positive mode (A) represents the [M+Na]^+^ adduct of the head group fragment and *m/z* 239 in the negative mode (B) represents the [M-H-H_2_O]^−^ fragment of the head group. The negative-mode MS/MS spectrum (B) confirms that the lipid contains FAs 16:0 and 18:1. Download FIG S4, PDF file, 0.04 MB. Copyright © 2017 Hines et al. 2017 Hines et al. This content is distributed under the terms of the Creative Commons Attribution 4.0 International license.

10.1128/mSphere.00492-17.9 TABLE S5  Individual lipid species altered in *C. striatum* strains W40308 (daptomycin susceptible) and W49297 (daptomycin resistant). Superscript letters: a, FAs determined from targeted MS/MS experiments in negative ionization mode; b, significance determined by Student's *t* test, *P* ≤ 0.05. Download TABLE S5, PDF file, 0.1 MB. Copyright © 2017 Hines et al. 2017 Hines et al. This content is distributed under the terms of the Creative Commons Attribution 4.0 International license.

## DISCUSSION

The results of the analyses of *E. faecalis*, *S. aureus*, and *C. striatum* indicate that the HILIC-IM-MS method is well suited for untargeted analysis of the bacterial lipidome. The major classes of glycerolipids, glycolipids, and glycerophospholipids present in typical Gram-positive bacteria, as well as some unknown and atypical lipid species, were resolved in <10 min in the HILIC dimension. In addition to retention time and accurate mass, CCS values from the IM-MS dimension provide additional validating information for the assignment of identifications to lipid species. For example, the CCS values measured for PG species of *E. faecalis* are <1% different from the CCS values previously reported for the same PG species (Table S2) (38). This study also reports 160 new lipid CCS values that can be used for future bacterial lipidome studies. When paired with biostatistics, the HILIC-IM-MS method offers reproducible relative quantitation of lipid signals to provide insight into the role of the bacterial lipidome in daptomycin resistance.

In comparison with the 2D TLC methods commonly used in the field (17, 34, 35), our HILIC-IM-MS method offers a number of advantages. First, our method allows the identification and quantification of individual molecular species of each lipid class. This is important because it provides additional insights into the molecular mechanisms underlying the lipidomic changes, such as the FA-dependent intensity profiles for all of the lipid classes shown in Fig. 3 (see additional discussion below). Second, the HILIC-IM-MS method enables identification and quantification of lipid classes that do not contain a phosphate group, such as FFA, DGDGs, and MGDGs, whereas the 2D TLC method does not. Third, our method offers high sensitivity and reproducibility to enable the discovery of smaller changes in individual lipid levels. Finally, the 12-min HILIC-IM-MS run offers much higher throughput than the labor-intensive workflow of 2D TLC.

The findings from the HILIC-IM-MS analysis of the S613-R712 *E. faecalis* strain pair confirm the results of a previous lipidomics study of the same *E. faecalis* strain pair, which reported that daptomycin resistance in R712 was associated with significantly reduced levels of PGs, as well as elevated levels of GP-DGDGs by a 2D TLC method (34). However, our analysis revealed, for the first time, additional significant changes in pPGs, lysyl-PGs, CLs, DGDGs, and MGDGs (Fig. 2). These lipidome alterations are likely the result of three known genetic mutations in R712, including mutations in two genes involved in phospholipid metabolism, those for glycerophosphoryl diester phosphodiesterase (GdpD) and CL synthase (Cls) (15). The third mutation was located in *liaF* (lipid II cycle-interfering antibiotic protein) of the three-component *liaFSR* cell envelope stress response regulatory system and may regulate lipid metabolism through DG kinase (DagK) (15, 49).

We observed FA-specific alterations in the abundances of lipid species in daptomycin-resistant *E. faecalis* and *S. aureus* N315. In *E. faecalis* strain R712, the significantly elevated DGDG species were all determined to contain at least one 18:1 FA. Interestingly, small increases in 18:1 FAs were also observed in the analysis of R712 by Mishra et al., but the differences were not statistically significant and could not be directly correlated with any specific class of lipid species (34). Other reports on daptomycin resistance in *E. faecalis* have observed a similar accumulation of exogenous 18:1 FAs and indicate that these changes in global FA content protect bacteria from membrane stress induced by daptomycin (50, 51). While the ability to incorporate exogenous 18:1 FA has been attributed to mutations in the LiaFSR system, recent reports have shown that this phenomenon can occur independently of LiaFSR mutations (51). Our results obtained with *E. faecalis* strain R712 suggest that this accumulation of 18:1 FA may affect selected lipid classes, such as DGDGs, rather than cause a global shift in FA content. These FA-specific changes underscore the importance of analyzing individual lipid species instead of examining each lipid class as a whole, as previously reported (33, 34).

We identified a mutation in daptomycin-resistant *S. aureus* N315 in the gene encoding YycG of the two-component regulatory system YycFG, which is thought to be orthologous to the *E. faecalis* LiaFSR system. The YycFG system is essential to *S. aureus* and has been shown to regulate nine genes involved in cell wall metabolism, such as the major autolysins AtlA and LytM (44, 45). Daptomycin-resistant *S. aureus* with mutations in *yycFG* frequently have thickened cell walls, which are thought to arise because of reduced autolysin activity as a result of the *yycFG* mutations (17, 23, 52, 53). In *Streptococcus pneumoniae*, YycFG has been shown to regulate FA biosynthesis genes and alter cell membrane composition (46, 47). While the role of YycFG in FA biosynthesis has not been confirmed in *S. aureus*, whether or not it contributes to the significant elevation of selected FFA species, particularly longer 19:0 to 21:0 FAs (Fig. 3H), in N315-D8 is worthy of further investigation.

Another genetic mutation identified in daptomycin-resistant N315 was located in the multiprotein resistance factor (*mprF*) gene. MprF has a 2-fold function of lysinylation of PGs and translocation of lysyl-PGs to the outer membrane leaflet. Previously reported mutations in *mprF* in daptomycin-resistant *S. aureus* are gain-of-function mutations (19, 54) that are thought to increase the net positive charge of the membrane by increasing the presence of positively charged lysyl-PGs in the outer membrane leaflet. The specific amino acid change predicted in MprF in N315-D8, L826F in the lysinylation domain, has been reported previously in both laboratory- and clinic-derived *S. aureus* strains with daptomycin resistance (MICs of 2 and 4 µg/ml, respectively) (22). Although *mprF* mutations are very common in daptomycin-resistant *S. aureus*, an increased abundance of lysyl-PGs and an increased positive surface charge are not consistently observed (19, 20, 54). In the present study, the abundance of lysyl-PGs was predominantly reduced in N315-D8, with the exception of minor increases in 34:0 and 36:0 lysyl-PGs. Such differential changes in individual species of lysyl-PGs have not been reported previously.

The observation of reduced lysyl-PGs in N315-D8, despite the presence of a gain-of-function *mprF* mutation, suggests that the metabolic consequence of the mutation in *pgsA* outweighs that of *mprF* mutation. PgsA catalyzes the transfer of glycerol-3-phosphate to CDP-DG to form PGP, which is the immediate precursor of PG (Fig. 4). Blocking of PG synthesis accounts for the reduced abundances of PGs and lipid products generated downstream of PGs (i.e., CLs and lysyl-PGs), as well as for the accumulation of lipid species upstream of PgsA, such as PAs. The similarities in the FA compositions of PAs and DGDGs suggest that the *pgsA* mutation also contributes to the elevated levels of DGDGs in N315-D8, possibly through a DG intermediate, as shown in Fig. 4. In comparison with the compositions of FFAs, it appears that significant increases in PAs and DGDGs with long total FA chains (34:0, 35:0, and 36:0) reflect the significant increases in the corresponding FFAs (15:0, 17:0, 19:0, 20:0, and 21:0) (see Table S4 for FA compositions). Interestingly, the levels of the long-chain PG 36:0 also increased in the resistant strain, which reflects changes in its FA composition: 15:0 and 21:0. This observation suggests that the mutation in *pgsA* does not inhibit the synthesis of PGs from all FAs. Such FA-dependent changes further emphasize the importance of analyzing individual lipid species within each class.

Mutations in *pgsA* have been reported previously in daptomycin-resistant *S. aureus* and *Bacillus subtilis*, but its effect on lipids upstream of PGs has not been evaluated (22, 33). Our results are supported by recent reports on daptomycin-resistant *S. oralis* and *S. mitis*, which showed that blocking of PG synthesis by mutations in the gene encoding CdsA (catalyzing the synthesis of CDP-DG) contributes to the accumulation of DGDG glycolipid species in addition to the expected accumulation of PAs (31, 32).

We observed a similar increase in glycolipid species in daptomycin-resistant *C. striatum* with a mutation in *pgsA2*. The cell wall architecture of *C. striatum* is distinct from that of *E. faecalis* and *S. aureus* (55). However, PIs and GlcADGs have a function homologous to that of DGDGs in that both serve as the membrane anchor of the cell wall polymers of *Corynebacterium* spp., lipomannan (LM) and lipoarabinomannan (LAM) (56, 57).

In the presence of a *pgsA* mutation, the accumulation of negatively charged PA species would seem to be disadvantageous for daptomycin-resistant bacteria, considering the mechanism of daptomycin to target regions of the bacterial membrane rich in negatively charged PG lipid species (7–9). However, the ability to shift the biosynthetic pathway away from PA synthesis and toward the synthesis of neutral glycolipid species, such as DGDGs, PIs, and GlcADGs, is likely protective against daptomycin, as it would reduce the negative charge of the cell membrane. The combination of increased glycolipids and decreased PGs due to mutation of *pgsA* could also affect the synthesis or abundance of the amphiphilic cell wall polymers, such as LTA (for *S. aureus* and *E. faecalis*), LM, and LAM (for *C. striatum*). PGs provide the glycerophosphate units to LTA, and glycolipids or their analogues (i.e., DGDGs in *E. faecalis* and *S. aureus* and GlcADG and PIs in *C. striatum*) act as the membrane anchors of LTA, LM, and LAM (Fig. 4). The contribution of increased glycolipid content to daptomycin resistance and the effect on related cell wall polymers are of interest for future investigation. To our knowledge, this is the first study of lipidomic changes associated with daptomycin resistance in *C. striatum*.

In summary, lipidomic changes associated with daptomycin resistance in several Gram-positive bacterial pathogens were characterized by a multidimensional HILIC-IM-MS lipidomic method that allows the analysis of glycerolipids, glycerophospholipids, and glycolipids with high throughput, sensitivity, and molecular specificity, providing additional insights into the underlying molecular mechanisms. Several alterations of the lipid profiles of daptomycin-resistant strains were found to be conserved in *S. aureus* and *C. striatum*, including PGs, CLs, amino-PGs, PAs, and glycolipids, which reflect the impact of *pgsA* mutation on the lipid biosynthetic pathway. However, the FA-dependent changes in individual species of each lipid class suggest that modification of FA composition, presumably by mutations in other genes (such as *yycFG*), also contributes significantly to lipid profiles. The characteristic changes in individual lipid species could be used as biomarkers for identifying the underlying resistance mechanisms. These results offer new insights into the role of membrane lipid composition in daptomycin resistance that may aid in developing new approaches for recovering daptomycin susceptibility or novel antibacterial therapeutics by targeting lipid metabolic pathways.

## MATERIALS AND METHODS

### Reagents.

High-performance LC grade solvents (water, acetonitrile, methylene chloride, chloroform, and methanol), ammonium acetate (Optima LC/MS), and sodium chloride were purchased from Thermo Fisher Scientific. The following extracts and standards were purchased from Avanti Polar Lipids: lysyl-PG 16:0 (LysylPG 840520), MGDG (Gal_1_DG 840523), DGDG (Gal_2_DG 840524), CLs (CLs 840012 and 841199), l-α-phosphatidic acid (PA 840101c), and l-α-PG (PG 841138c). The following FFA and diacylglyceride standards were purchased from Nu Chek Prep: pentadecanoic acid (FA 15:0, N-15-A), hexadecanoic acid (FA 16:0, N-16-A), heptadecanoic acid (FA 17:0, N-17-A), octadecanoic acid (FA 18:0, N-18-A), nonadecanoic acid (FA 19:0, N-19-A), eicosanoic acid (FA 20:0, N-20-A), heneicosanoic acid (FA 21:0, N-21-A), 1,3-dimyristoyldiacylglyceride (1,3-DG 14:0, D-142), 1,3-dipalmitoyldiacylglycerol (1,3-DG 16:0, D-152), 1,3-distearoyldiacylglyceride (1,3-DG 18:0, D-162), 1,3-dioleoyldiacylglyceride (1,3-DG 18:1, D-237), *cis*-1,3-dilinoleoyldiacylglyceride (*cis*-1,3-DG 18:2, D-252), 1,3-diarachidoyldiacylglyceride (1,3-DG 20:0, D-172), and 1,3-diarachidonoyldiacylglyceride (1,3-DG 20:4, D-297). Stock solutions of lipid standards and standard extracts were prepared at 1 mM in chloroform, from which a 5 µM mixture in 2:1 acetonitrile-methanol was prepared for analysis.

### Bacterial strains and *in vitro* selection of daptomycin nonsusceptibility.

The clinically derived daptomycin-susceptible and -resistant strain pairs of *E. faecalis* (S613 and R712) and *C. striatum* (W40308 and W49297) used in this study have been described previously (15, 40). We used *in vitro* passage techniques to select a daptomycin-nonsusceptible mutant of well-characterized laboratory strain N315 by methods similar to those described previously (43). Briefly, cultures of N315 were prepared in brain heart infusion (BHI) broth supplemented with 50 mg/liter elemental calcium (BHI-50), exposed to the MIC of daptomycin, and incubated at 37°C with shaking. Visible growth was diluted 1:100 in BHI-50 supplemented with daptomycin at a concentration twice as high as the previous concentration or 2 µg/ml higher than the previous concentration, whichever was lower. This process was repeated for 30 days before an isolate capable of growing in 12 mg/liter daptomycin was selected. The resulting isolate was subjected to susceptibility testing by broth microdilution before and after 5 days of serial passage on antibiotic-free medium and confirmed to have a stable MIC of 8 mg/liter. Each isolate was grown in triplicate in 50 ml of BHI broth and for 24 h, pelleted by centrifugation, dried in a vacuum concentrator (Thermo Fisher Savant), weighed, and stored at −80°C until analysis. The average dry pellet weights per group were as follows: N315, 100 ± 3.7 mg; N315-D8, 85.0 ± 0.4 mg; S613, 82.4 ± 8.8 mg; R712, 67.6 ± 1.3 mg; W40308, 176.1 ± 5.5 mg; W49297, 41.4 ± 0.2 mg.

### Lipid extraction.

Lipid extraction was carried out by the method of Bligh and Dyer as described elsewhere (41, 42, 58). Briefly, 1 ml of water was added to the pelleted bacteria in 10-ml glass centrifuge tubes and the samples were sonicated in an ice bath for 30 min to dislodge the dried pellets. A chilled solution of chloroform and methanol (1:2, 4 ml) was added to each tube. The samples were vortexed for 5 min, and a 1.25-ml volume each of chilled chloroform and chilled water was added to the tubes with brief vortexing between solvent additions. The samples were centrifuged for 10 min at 10°C and 1,100 × *g* to separate the aqueous and organic layers. The organic phase was transferred into 10-ml glass centrifuge tubes and dried in a vacuum concentrator. Dried extracts were reconstituted in 500 µl of 1:1 chloroform-methanol. For analysis, 10 µl of the lipid extract was transferred to an LC vial, dried under Ar, and reconstituted to 200 µl with 2:1 acetonitrile-methanol. We found that the choice of reconstitution solvent for HILIC-IM MS analysis significantly impacted the ability to observe CLs. The choice of 2:1 acetonitrile-methanol sufficiently solubilized CLs while maintaining typical HILIC peak shapes and retention times. For N315, additional samples were prepared as 1:2 dilutions for the analysis of CLs and PAs.

### LC.

As described previously, chromatographic separations were performed with a Phenomenex Kinetex HILIC column (2.1 by 100 mm, 1.7 µm) on a Waters Acquity FTN UPLC (Waters Corp., Milford, MA) (38). The column temperature was maintained at 40°C, and the sample chamber was maintained at 6°C. The mobile phases for HILIC separation consisted of (i) 50% acetonitrile–50% water with 5 mM ammonium acetate and (ii) 95% acetonitrile–5% water with 5 mM ammonium acetate. A flow rate of 0.5 ml/min was used to achieve the following linear gradient: 0 to 1 min, 100% B; 4 min, 90% B; 7 to 8 min, 70% B; 9 to 12 min, 100% B. The injection volumes used for positive- and negative-mode analyses were 5 and 10 µl, respectively. The lipid species of interest eluted between 0.4 and 9.0 min, and the column was allowed to equilibrate to the initial conditions for 3 min (from 9 to 12 min) prior to the next injection.

### IM-MS.

IM-MS analysis was performed on a Waters Synapt G2-Si HDMS (Waters Corp., Milford, MA) equipped with an electrospray ionization (ESI) source. ESI capillary voltages of +2.5 and −2.0 kV were used for positive- and negative-mode analyses, respectively. Other ESI conditions were as follows: sampling cone voltage, 40 V; extraction cone voltage, 80 V; source temperature, 150°C; desolvation temperature, 500°C; cone gas flow rate, 10 liters/h; desolvation gas flow rate, 1,000 liters/h. Mass calibration was performed with sodium formate for the *m/z* range of 50 to 1,200. As previously described, CCS calibration was performed with a set of PC and PE CCS standards (38, 39). IM separation was performed with a traveling-wave velocity of 500 m/s and a height of 40 V. Untargeted MS/MS was performed with a collision energy ramp of 35 to 45 eV applied to the transfer region of the instrument. Data were acquired over an *m/z* range of 50 to 1,200 with a 1-s scan time. Leucine enkephalin was acquired as a lockspray signal for postacquisition correction of *m/z* and drift time. Additional targeted MS/MS experiments were performed with a collision energy ramp of 35 to 45 eV to determine the FA contents of selected lipid species (in negative mode) and aid in the identification of unknown lipids (in positive and negative modes).

### Data analysis.

Data alignment, peak detection, and normalization were performed in Progenesis QI (Nonlinear Dynamics). The chromatographic region from 0.4 to 9.0 min was considered for peak detection. The reference sample for alignment was selected by Progenesis QI from the pooled quality control samples, and data were normalized to the bacterial pellet weights. The resulting features were filtered by analysis of variance (*P* ≤ 0.05). Student's *t* tests for two samples were performed by using a two-tailed distribution and equal variance. When possible, identifications were made against the METLIN database within a mass accuracy of 15 ppm (59, 60). For lipid species not found in common lipidomics databases, in-house databases were generated with ChemDraw (DGDGs and lysyl-PGs; PerkinElmer) or LipidPioneer (MGDGs and CLs) (61). CCS values for lipid standard extracts were obtained by using the DriftScope v2.8 (Waters Corp.) chromatographic peak detection algorithm with lock mass correction.

### Whole-genome sequencing.

Whole-genome sequencing of the *C. striatum* strain pair (W40308 and W49297) and the N315 derivative was performed with the MiSeq platform (Illumina, San Diego, CA) as previously described (62), to average read depths of 83×, 114×, and 100×, respectively. Sequences for *E. faecalis* strains S613 and R712 have been previously published (15). For analysis of *S. aureus*, sequence reads were analyzed against the N315 reference genome (GenBank accession number BA000018) (63) as previously described (62), with sequence variants annotated by using SnpEFF (64). For analysis of *C. striatum*, the susceptible strain was first *de novo* assembled by using ABySS (65) and annotated by using PROKKA (66). Sequence reads from both *C. striatum* isolates were then mapped to the *de novo* assembly and subjected to variant calling as previously described (62), with sequence variants annotated by using custom scripts.

### Data availability.

Whole-genome sequencing data from this study are available in the NCBI Sequence Read Archive (http://www.ncbi.nlm.nih.gov/sra) under study accession number PRJNA379970.
