# Supplementary material for: Characterization of the Mechanisms of Daptomycin Resistance among Gram-Positive Bacterial Pathogens by Multidimensional Lipidomics
Source: mSphere. 2017 Dec 13;2(6):e00492-17. doi: 10.1128/mSphere.00492-17 (PMC5729219; doi:10.1128/mSphere.00492-17)
Supplement: TABLE S1 [file sph006172426st1.pdf]

Negative Mode CCS Values						
**Identification**	**Ret. Time (min)**	**Adduct**	***m/z***	**Accuracy (ppm)**	**CCS_{cal} [a] (Å^2)**	**RSD (%)**
Gal_1DG 16:3-18:3	0.60	$[M-H]^-$	745.490	0.9	269.9	0.0
Gal_1DG 18:3-18:3	0.59	$[M-H]^-$	773.520	0.0	276.2	0.0
Gal_1DG 16:3-18:3	0.63	$[M+Cl]^-$	781.466	0.8	273.7	0.1
Gal_1DG 16:3-18:3	0.60	$[M+CH3COO]^-$	805.512	2.7	280.3	0.1
Gal_1DG 18:3-18:3	0.60	$[M+Cl]^-$	809.493	-4.6	278.5	0.0
Gal_1DG 18:3-18:3	0.59	$[M+CH3COO]^-$	833.541	-1.2	286.3	0.1
Gal_2DG 16:3-18:4	0.64	$[M-H]^-$	905.524	-2.7	297.1	0.1
Gal_2DG 16:1-18:3	0.65	$[M-H]^-$	911.572	-0.9	301.4	0.1
Gal_2DG 18:4-18:3	0.65	$[M-H]^-$	933.557	-0.3	303.2	0.1
Gal_2DG 18:4-18:3	0.65	$[M+CH_3COO]^-$	993.578	-0.4	311.5	0.1
Gal_2DG 16:3-18:4	0.66	$[M+Cl]^-$	941.506	2.9	299.1	0.1
Gal_2DG 16:3-18:4	0.66	$[M+CH_3COO]^-$	965.550	2.3	305.3	0.1
Gal_2DG 18:4-18:3	0.68	$[M+Cl]^-$	969.535	0.7	304.7	0.1
Gal_2DG 16:1-18:3	0.69	$[M+Cl]^-$	947.550	-0.3	303.2	0.1
Gal_2DG 16:3-18:3	1.25	$[M-H]^-$	907.542	0.2	296.1	0.0
Gal_2DG 16:3-18:3	1.26	$[M+Cl]^-$	943.519	0.3	299.9	0.0
Gal_2DG 16:3-18:3	1.27	$[M+CH_3COO]^-$	967.562	-0.6	305.8	0.1
Gal_2DG 18:3-18:3	1.32	$[M+Cl]^-$	971.550	0.0	305.9	0.0
Gal_2DG 16:0-18:2	1.33	$[M+CH_3COO]^-$	975.621	-4.7	312.7	0.1
Gal_2DG 18:3-18:3	1.33	$[M+CH_3COO]^-$	995.594	-0.8	313.7	0.0
Gal_2DG 18:3-18:3	1.33	$[M-H]^-$	935.573	0.2	303.3	0.0
Gal_2DG 16:0-18:2	1.33	$[M+Cl]^-$	951.573	-8.5	304.9	0.0
Gal_2DG 16:0-18:2	1.33	$[M-H]^-$	915.602	-3.2	301.9	0.0
Gal_2DG 16:0-18:3	1.33	$[M+Cl]^-$	949.564	-1.6	304.4	0.0
Gal_2DG 16:0-18:3	1.33	$[M-H]^-$	913.589	-0.4	301.2	0.0
Gal_2DG 16:0-18:3	1.33	$[M+CH_3COO]^-$	973.604	-6.5	311.9	0.1
LysylPG 16:0-16:0	7.54	$[M-H]^-$	849.597	-0.6	291.5	0.1
**Positive Mode CCS Values**						
**Identification**	**Ret. Time (min)**	**Adduct**	***m/z***	**Accuracy (ppm)**	**CCS_{cal} [a] (Å^2)**	**RSD (%)**
Gal_1DG 16:3-18:3	0.59	$[M+NH_4]^+$	764.540	11.9	276.0	0.1
Gal_1DG 16:1-18:3	0.58	$[M+NH_4]^+$	768.560	-3.5	281.2	0.1
Gal_1DG 16:3-18:3	0.58	$[M+Na]^+$	769.494	9.6	273.1	0.0
Gal_1DG 16:1-18:3	0.57	$[M+Na]^+$	773.516	-3.1	278.6	0.3
Gal_1DG 16:3-18:3	0.58	$[M+K]^+$	785.462	2.3	273.6	0.0
Gal_1DG 18:3-18:3	0.58	$[M+NH_4]^+$	792.565	3.4	284.1	0.1
Gal_1DG 18:3-18:3	0.57	$[M+Na]^+$	797.520	2.8	281.0	0.0
Gal_1DG 18:3-18:3	0.57	$[M+K]^+$	813.494	2.7	282.5	0.1
Gal_2DG 16:3-18:4	0.69	$[M+NH_4]^+$	924.570	1.7	294.5	0.1
Gal_2DG 16:3-18:4	0.70	$[M+Na]^+$	929.526	2.0	292.9	0.1
Gal_2DG 16:1-18:3	0.72	$[M+NH_4]^+$	930.617	2.1	303.5	0.1
Gal_2DG 16:3-18:4	0.72	$[M+K]^+$	945.511	13.6	293.5	0.1

Gal_2DG 18:4-18:3	0.72	$[M+NH_4]^+$	952.600	0.4	301.3	0.1
Gal_2DG 18:4-18:3	0.72	$[M+Na]^+$	957.558	2.8	299.5	0.1
Gal_2DG 18:4-18:3	0.74	$[M+K]^+$	973.542	13.4	300.5	0.1
Gal_2DG 16:1-18:3	0.74	$[M+Na]^+$	935.576	5.2	301.1	0.0
Gal_2DG 16:3-18:3	1.34	$[M+Na]^+$	931.543	3.8	294.9	0.0
Gal_2DG 16:3-18:3	1.35	$[M+NH_4]^+$	926.588	4.2	296.3	0.0
Gal_2DG 18:3-18:3	1.39	$[M+NH_4]^+$	954.619	3.4	303.2	0.0
Gal_2DG 18:3-18:3	1.40	$[M+Na]^+$	959.573	2.7	301.9	0.0
Gal_2DG 16:0-18:2	1.40	$[M+NH_4]^+$	934.647	0.4	307.5	0.0
Gal_2DG 16:0-18:2	1.41	$[M+Na]^+$	939.601	-1.4	306.5	0.0
Gal_2DG 16:0-18:3	1.41	$[M+NH_4]^+$	932.636	4.8	305.3	0.0
Gal_2DG 16:0-18:3	1.43	$[M+Na]^+$	937.589	2.5	303.8	0.0
Gal_2DG 16:3-18:3	1.50	$[M+K]^+$	947.527	14.7	295.2	0.0
Gal_2DG 18:3-18:3	1.55	$[M+K]^+$	975.563	18.6	302.5	0.0
LysylPG 16:0-16:0	7.61	$[M+H]^+$	851.613	1.5	297.0	0.0
