# Supplementary material for: Characterization of the Mechanisms of Daptomycin Resistance among Gram-Positive Bacterial Pathogens by Multidimensional Lipidomics
Source: mSphere. 2017 Dec 13;2(6):e00492-17. doi: 10.1128/mSphere.00492-17 (PMC5729219; doi:10.1128/mSphere.00492-17)
Supplement: TABLE S2 [file sph006172426st2.pdf]

E. faecalis S613 and R712								
**Putative ID**	**Ret. Time (min)**	**Adduct**	***m/z***	**Accuracy (ppm)**	**CCS_{cal} [a] ($Å^2$)**	**RSD (%)**	**CCS_{ref} ($Å^2$)**	**ΔCCS (% diff)**
DGDG 28:1	1.14	$[M+Na]^+$	857.526	2.0	289.5	0.09		
DGDG 30:2	1.09	$[M+NH_4]^+$	878.585	1.5	295.4	0.01		
DGDG 30:1	1.10	$[M+NH_4]^+$	880.603	3.5	298.4	0.02		
DGDG 30:0	1.09	$[M+NH_4]^+$	882.614	-1.2	302.4	0.02		
DGDG 30:2	1.12	$[M+Na]^+$	883.537	-2.6	291.7	0.05		
DGDG 30:1	1.11	$[M+Na]^+$	885.557	1.8	295.5	0.02		
DGDG 30:0	1.12	$[M+Na]^+$	887.569	-1.8	299.6	0.10		
DGDG 30:1	1.12	$[M+K]^+$	901.532	2.7	298.1	0.02		
DGDG 32:2	1.06	$[M+NH_4]^+$	906.619	4.3	302.2	0.04		
DGDG 32:1	1.07	$[M+NH_4]^+$	908.637	6.1	305.4	0.04		
DGDG 32:0	1.06	$[M+NH_4]^+$	910.642	-5.1	307.4	0.03		
DGDG 32:2	1.10	$[M+Na]^+$	911.573	2.6	298.0	0.04		
DGDG 32:1	1.10	$[M+Na]^+$	913.587	1.0	302.5	0.02		
DGDG 32:0	1.11	$[M+Na]^+$	915.597	-6.0	304.7	0.05		
DGDG 32:1	1.12	$[M+K]^+$	929.563	3.1	305.1	0.01		
DGDG 34:3	1.03	$[M+NH_4]^+$	932.631	0.1	305.8	0.07	305.3 [c]	0.2
DGDG 34:2	1.03	$[M+NH_4]^+$	934.653	6.5	309.0	0.06	307.5 [c]	0.5
DGDG 34:1	1.03	$[M+NH_4]^+$	936.667	5.0	311.6	0.06		
DGDG 34:2	1.02	$[M+Na]^+$	939.606	4.0	305.2	0.03	306.5 [c]	-0.4
DGDG 34:1	1.02	$[M+Na]^+$	941.618	0.6	309.0	0.06		
DGDG 34:1	1.18	$[M+K]^+$	957.604	12.4	311.3	0.03		
DGDG 35:2	1.03	$M+NH_4]^+$	948.664	2.4	315.0	0.08		
DGDG 35:2	1.02	$[M+Na]^+$	953.620	2.6	311.5	0.06		
DGDG 35:1	1.04	$M+NH_4]^+$	950.675	-2.4	317.4	0.12		
DGDG 36:2	1.01	$[M+NH_4]^+$	962.682	4.4	315.2	0.07		
DGDG 36:2	0.99	$[M+Na]^+$	967.635	1.9	312.4	0.08		
DGDG 36:2	0.99	$[M+K]^+$	983.610	2.5	315.3	0.11		
PG p31:2	1.40	$[M-H]^-$	687.461	-0.6	256.8	0.02		
PG p33:2	1.37	$[M-H]^-$	715.493	2.7	263.4	0.01		
PG p34:3	1.35	$[M-H]^-$	727.492	1.4	264.8	0.02		
PG p34:2	1.37	$[M-H]^-$	729.508	1.4	266.6	0.00		
PG 28:1	2.42	$[M-H]^-$	663.424	-1.1	251.2	0.13		
PG 28:0	2.43	$[M-H]^-$	665.438	-2.9	253.1	0.15		
PG 30:2	2.37	$[M-H]^-$	689.440	-0.1	255.0	0.10		
PG 30:1	2.40	$[M-H]^-$	691.457	2.3	257.1	0.03		
PG 30:0	2.40	$[M-H]^-$	693.470	-2.0	258.9	0.03		
PG 31:2	2.28	$[M-H]^-$	703.456	0.0	257.9	0.07		
PG 31:1	2.38	$[M-H]^-$	705.471	-0.4	260.2	0.04		
PG 32:2	2.30	$[M-H]^-$	717.473	2.4	261.4	0.06	261.0 [b]	0.1
PG 32:1	2.32	$[M-H]^-$	719.489	2.8	263.3	0.04	262.5 [b]	0.3
PG 32:0	2.32	$[M-H]^-$	721.501	-2.5	264.9	0.08	263.5 [b]	0.5
PG 33:2	2.29	$[M-H]^-$	731.487	0.4	264.5	0.02		
PG 33:1	2.31	$[M-H]^-$	733.503	1.2	267.0	0.06		
PG 34:2	2.25	$[M-H]^-$	745.503	0.3	268.1	0.03	267.4 [b]	0.2

PG 34:1	2.27	[M-H]$^-$	747.518	0.0	269.9	0.03	268.9 [b]	0.4
PG 34:0	2.26	[M-H]$^-$	749.529	-6.4	270.8	0.08	269.0 [b]	0.7
PG 35:2	2.23	[M-H]$^-$	759.519	1.6	271.4	0.03		
PG 35:1	2.26	[M-H]$^-$	761.535	1.2	273.2	0.07		
PG 36:2	2.19	[M-H]$^-$	773.533	-0.5	274.9	0.07	274.3 [b]	0.2
PG 36:1	2.19	[M-H]$^-$	775.544	-7.6	275.7	0.07	275.7 [b]	0.0
PG 37:1	2.18	[M-H]$^-$	787.549	-1.3	278.0	0.05		
LysylPG 30:1	7.73	[M-H]$^-$	821.569	-4.7	287.0	0.01		
LysylPG 30:0	7.75	[M+H]$^+$	823.584	-4.2	290.4	0.02		
LysylPG 32:2	7.68	[M+H]$^+$	847.589	-9.6	290.8	0.04		
LysylPG 32:1	7.70	[M+H]$^+$	849.601	-5.2	293.5	0.03		
LysylPG 34:2	7.63	[M+H]$^+$	875.625	-15.3	298.4	0.09		
LysylPG 34:1	7.66	[M+H]$^+$	877.632	-4.9	299.6	0.03		
LysylPG 35:2	7.63	[M+H]$^+$	889.632	-4.8	300.8	0.02		
LysylPG 35:1	7.66	[M+H]$^+$	891.647	-4.0	303.5	0.01		
LysylPG 36:2	7.61	[M+H]$^+$	903.647	-3.7	302.8	0.00		
***S. aureus* N315 and N315-D8**								
**Putative ID**	**Ret. Time (min)**	**Adduct**	***m/z***	**Accuracy (ppm)**	**CCS_{cal} [a] (Å^2)**	**RSD (%)**	**CCS_{ref} (Å^2)**	**ΔCCS (% diff)**
DGDG 29:0	1.17	[M+NH$_4$]$^+$	868.600	0.6	298.3	0.03		
DGDG 30:0	1.17	[M+NH$_4$]$^+$	882.621	6.3	301.8	0.04		
DGDG 30:0	1.17	[M+Na]$^+$	887.573	2.0	299.6	0.03		
DGDG 31:0	1.15	[M+NH$_4$]$^+$	896.633	2.3	305.4	0.05		
DGDG 31:0	1.15	[M+Na]$^+$	901.587	0.2	303.4	0.05		
DGDG 32:0	1.14	[M+NH$_4$]$^+$	910.652	5.7	308.5	0.08		
DGDG 32:0	1.14	[M+Na]$^+$	915.603	1.3	306.7	0.07		
DGDG 33:0	1.11	[M+NH$_4$]$^+$	924.665	2.7	311.5	0.06		
DGDG 33:0	1.11	[M+Na]$^+$	929.618	0.4	309.0	0.07		
DGDG 34:0	1.10	[M+NH$_4$]$^+$	938.680	2.2	314.2	0.08		
DGDG 34:0	1.09	[M+Na]$^+$	943.634	0.1	311.8	0.08		
DGDG 35:0	1.07	[M+NH$_4$]$^+$	952.695	1.5	317.1	0.06		
DGDG 35:0	1.07	[M+Na]$^+$	957.650	0.4	314.2	0.07		
DGDG 36:0	1.05	[M+NH$_4$]$^+$	966.709	-0.1	326.8	0.01		
PG p29:1	1.59	[M-H]$^-$	661.445	1.4	252.2	0.04		
PG p30:1	1.55	[M-H]$^-$	675.461	1.5	255.3	0.04		
PG p31:1	1.53	[M-H]$^-$	689.477	2.3	258.5	0.03		
PG p32:1	1.50	[M-H]$^-$	703.492	1.3	262.1	0.07		
PG p33:1	1.49	[M-H]$^-$	717.508	1.4	265.1	0.07		
PG p34:1	1.45	[M-H]$^-$	731.523	0.8	268.6	0.03		
PG 29:0	2.41	[M-H]$^-$	679.457	2.2	255.2	0.02		
PG 30:0	2.39	[M-H]$^-$	693.480	13.1	258.7	0.05		
PG 31:0	2.33	[M-H]$^-$	707.492	7.3	262.0	0.07		
PG 32:0	2.30	[M-H]$^-$	721.512	13.6	265.1	0.08	263.5 [b]	0.6
PG 33:0	2.25	[M-H]$^-$	735.524	7.5	268.6	0.07		
PG 34:0	2.21	[M-H]$^-$	749.538	5.6	271.6	0.05	269.0 [b]	1.0
PG 35:0	2.16	[M-H]$^-$	763.552	2.6	275.0	0.04		
PG 36:0	2.13	[M-H]$^-$	777.565	-0.1	277.9	0.06		

PA 30:0	6.46	$[M-H]^-$	619.434	0.5	244.9	0.01		
PA 31:0	6.45	$[M-H]^-$	633.450	0.0	248.2	0.03		
PA 32:0	6.44	$[M-H]^-$	647.467	-1.7	251.1	0.01		
PA 33:0	6.42	$[M-H]^-$	661.481	0.6	254.5	0.02		
PA 34:0	6.43	$[M-H]^-$	675.499	-2.2	257.6	0.01		
PA 35:0	6.43	$[M-H]^-$	689.513	-0.9	261.0	0.02		
PA 36:0	6.42	$[M-H]^-$	703.529	-0.3	264.1	0.01		
LysylPG 28:0	7.70	$[M+H]^+$	795.550	1.3	284.7	0.04		
LysylPG 29:0	7.66	$[M+H]^+$	809.566	0.7	287.8	0.03		
LysylPG 30:0	7.64	$[M+H]^+$	823.582	1.9	290.9	0.01		
LysylPG 31:0	7.60	$[M+H]^+$	837.598	2.0	293.9	0.03		
LysylPG 32:0	7.57	$[M+H]^+$	851.613	0.9	296.8	0.02	297.0 [c]	-0.1
LysylPG 33:0	7.54	$[M+H]^+$	865.629	1.0	299.8	0.03		
LysylPG 34:0	7.52	$[M+H]^+$	879.644	0.8	302.5	0.02		
LysylPG 35:0	7.49	$[M+H]^+$	893.661	1.7	305.4	0.04		
LysylPG 36:0	7.59	$[M+H]^+$	907.677	-9.2	308.7	0.02		
***C. striatum* W40308 and W49297**								
**Putative ID**	**Ret. Time (min)**	**Adduct**	***m/z***	**Accuracy (ppm)**	**CCS$_{cal}$ [a] (Å^2)**	**RSD (%)**	**CCS$_{ref}$ (Å^2)**	**ΔCCS (% diff)**
1.36_834.5291n	1.45	$[M+Na]^+$	857.523		292.8	0.01		
1.36_834.5291n	1.53	$[M+K]^+$	873.497		294.1	0.02		
GlcADG 16:0-18:1	2.25	$[M+NH_4]^+$	788.596	-10.4	290.1	0.07		
GlcADG 16:0-18:1	2.27	$[M+Na]^+$	793.551	-9.6	288.1	0.07		
GlcADG 16:0-16:0	2.33	$[M+NH_4]^+$	762.578	-6.6	286.0	0.07		
GlcADG 16:0-16:0	2.35	$[M+Na]^+$	767.532	-5.6	282.6	0.03		
PI 16:0-18:1	3.74	$[M+Na]^+$	859.540	-11.1	295.4	0.06		
PI 16:0-16:0	3.82	$[M+Na]^+$	833.521	-7.3	289.5	0.03		
AlaPG 34:1	4.51	$[M+H]^+$	820.581	-13.5	291.7	0.07		
AlaPG 34:1	4.50	$[M+Na]^+$	842.560	-9.9	294.3	0.10		
AlaPG 34:1	4.49	$[M+K]^+$	858.530	-4.9	293.6	0.11		
0.90_934.7546m/z	0.97	$[M-H]^-$	934.759		318.2	0.02		
1.36_834.5291n	1.35	$[M-H]^-$	833.527		282.8	0.02		
PG 16:0-18:1	2.06	$[M-H]^-$	747.521	-4.0	269.2	0.03	268.9 [b]	0.1
PG 16:0-16:0	2.15	$[M-H]^-$	721.505	-3.0	264.5	0.01	263.5 [b]	0.4
GlcADG 16:0-18:1	2.29	$[M-H]^-$	769.554	-8.4	272.7	0.03		
GlcADG 16:0-16:0	2.38	$[M-H]^-$	743.530	2.4	267.9	0.02		
PI 16:0-18:1	3.71	$[M-H]^-$	835.541	-7.7	285.5	0.02	283.7 [b]	0.6
PI 16:0-16:0	3.75	$[M-H]^-$	809.523	-5.3	280.9	0.05		
PA 16:0-18:1	6.28	$[M-H]^-$	673.484	-3.9	256.3	0.01	254.7 [b]	0.6
PA 16:0-16:0	6.49	$[M-H]^-$	647.469	-5.7	251.4	0.00		
