# Supplementary material for: Characterization of the Mechanisms of Daptomycin Resistance among Gram-Positive Bacterial Pathogens by Multidimensional Lipidomics
Source: mSphere. 2017 Dec 13;2(6):e00492-17. doi: 10.1128/mSphere.00492-17 (PMC5729219; doi:10.1128/mSphere.00492-17)
Supplement: TABLE S3 [file sph006172426st3.pdf]

Putative ID	Ret. Time (min)	*m/z*	Adduct	Fatty Acids [a]	Accuracy (ppm)	S613 (x10^3)	Fold (R712/S613)	*P*-value [b]
MGDG 32:2	0.55	749.521	$[M+Na]^+$		4.7	11.2 ± 0.6	1.4	0.0016
MGDG 32:0	0.59	748.592	$[M+NH_4]^+$		-1.9	18.8 ± 0.6	1.2	0.0083
MGDG 34:2	0.59	772.596	$[M+NH_4]^+$		2.9	15 ± 0.3	1.2	0.015
MGDG 34:1	0.52	779.570	$[M+Na]^+$		7.4	198.5 ± 8.6	1.0	0.42
MGDG 35:1	0.59	788.624	$[M+NH_4]^+$		-0.7	14.4 ± 0.2	1.4	0.0069
MGDG 36:2	0.59	800.626	$[M+NH_4]^+$		2.0	20.6 ± 0.5	1.3	0.0072
MGDG 37:2	0.59	814.635	$[M+NH_4]^+$		-6.3	2.4 ± 0.1	1.8	3.0 x 10^{-4}
DGDG 30:1	1.05	880.601	$[M+NH_4]^+$		1.3	39.1 ± 4.2	1.4	0.010
DGDG 32:2	1.02	906.616	$[M+NH_4]^+$	18:1, 16:1, 14:1	1.0	46.6 ± 4.5	1.3	0.012
DGDG 32:1	1.02	908.633	$[M+NH_4]^+$	14:0, 18:1, 16:0, 16:1	1.6	218.1 ± 25.3	1.2	0.054
DGDG 34:1	0.98	936.665	$[M+NH_4]^+$	18:1, 16:0	3.3	674.1 ± 75.7	1.7	9.4 x 10^{-4}
DGDG 35:2	0.93	953.620	$[M+Na]^+$		2.4	115.7 ± 12.7	0.9	0.21
DGDG 35:1	0.98	950.677	$[M+NH_4]^+$	19:1, 16:0	-1.0	137.1 ± 14.5	1.0	0.83
DGDG 36:2	0.93	962.679	$[M+NH_4]^+$	18:1	1.2	148.4 ± 15.8	2.4	9.3 x 10^{-5}
DGDG 37:2	0.93	976.692	$[M+NH_4]^+$		-1.8	20.9 ± 2.2	1.8	7.7 x 10^{-4}
DGDG 38:2	0.93	990.704	$[M+NH_4]^+$		-5.4	2.8 ± 0.3	2.3	2.9 x 10^{-4}
PG p31:2	1.36	687.464	$[M-H]^-$		5.5	10.2 ± 0.8	0.7	0.0063
PG p33:2	1.33	715.491	$[M-H]^-$		-0.6	27.2 ± 0.9	0.8	0.0083
PG p34:3	1.33	727.492	$[M-H]^-$		0.4	3.6 ± 0.1	0.6	9.3 x 10^{-4}
PG p34:2	1.33	729.506	$[M-H]^-$		-1.6	11.1 ± 0.6	0.8	0.024
PG p35:3	1.29	741.506	$[M-H]^-$		-2.1	4.7 ± 0.1	0.7	0.0022
PG 26:0	2.38	637.407	$[M-H]^-$		-2.9	1.3 ± 0.1	0.3	1.6 x 10^{-4}
PG 28:1	2.29	663.423	$[M-H]^-$		-1.7	5.9 ± 0.4	0.3	1.8 x 10^{-4}
PG 28:0	2.29	665.439	$[M-H]^-$		-2.0	9.9 ± 0.7	0.2	1.4 x 10^{-4}
PG 30:2	2.22	689.441	$[M-H]^-$		1.5	6.7 ± 0.3	0.4	2.4 x 10^{-5}
PG 30:1	2.22	691.456	$[M-H]^-$		0.1	35.8 ± 1.4	0.4	3.2 x 10^{-5}
PG 30:0	2.22	693.471	$[M-H]^-$		-0.7	37.9 ± 2.5	0.4	3.2 x 10^{-4}
PG 32:2	2.14	717.481	$[M-H]^-$	14:1, 16:1, 18:1	13.5	32.7 ± 0.8	0.6	2.2 x 10^{-4}
PG 32:1	2.14	719.489	$[M-H]^-$	14:0, 16:0, 16:1, 18:1	2.5	138.6 ± 4.7	0.5	4.0 x 10^{-5}
PG 33:2	2.10	731.496	$[M-H]^-$		12.7	20.7 ± 0.5	0.5	3.1 x 10^{-5}
PG 33:1	2.14	733.505	$[M-H]^-$		3.5	88.6 ± 3.4	0.5	1.9 x 10^{-4}
PG 34:2	2.07	745.515	$[M-H]^-$	16:1, 18:1	16.9	81.7 ± 1.3	0.7	0.0013
PG 34:1	2.07	747.521	$[M-H]^-$	16:0, 18:1	4.1	261.2 ± 8.3	0.5	5.9 x 10^{-5}
PG 35:2	2.01	759.519	$[M-H]^-$		0.5	66.8 ± 1.8	0.5	3.4 x 10^{-5}
PG 35:1	2.07	761.535	$[M-H]^-$	16:0, 19:1	2.0	184.1 ± 5.8	0.6	2.8 x 10^{-4}
PG 36:2	2.01	773.534	$[M-H]^-$	18:1	0.7	89.9 ± 1.4	0.4	4.7 x 10^{-6}
CL 60:2	4.74	645.430	$[M-2H]^{-2}$		-3.4	1.2 ± 0.1	0.4	3.8 x 10^{-4}
CL 60:1	4.81	646.437	$[M-2H]^{-2}$		-4.2	1.7 ± 0.2	0.4	8.5 x 10^{-4}
CL 62:3	4.65	658.440	$[M-2H]^{-2}$		0.1	1.4 ± 0	0.4	4.5 x 10^{-5}
CL 62:2	4.74	659.446	$[M-2H]^{-2}$		-2.7	8.5 ± 0.4	0.4	6.4 x 10^{-5}

CL 64:3	4.70	672.455	$[M-2H]^{-2}$		-0.8	4.6 ± 0	0.4	1.5×10^{-6}
CL 64:2	4.81	673.462	$[M-2H]^{-2}$		-2.3	16.6 ± 0.7	0.4	4.7×10^{-5}
CL 65:3	4.81	679.474	$[M-2H]^{-2}$		16.2	10.6 ± 0.2	0.7	1.3×10^{-4}
CL 66:4	4.70	685.465	$[M-2H]^{-2}$		2.8	3.3 ± 0.1	0.4	7.7×10^{-6}
CL 66:2	4.86	687.477	$[M-2H]^{-2}$	18:1, 16:0, 16:1, 14:0	-2.2	35.4 ± 2	0.3	9.9×10^{-5}
CL 67:4	4.81	692.483	$[M-2H]^{-2}$		16.7	5.7 ± 0.2	0.4	7.2×10^{-5}
CL 67:3	4.89	693.485	$[M-2H]^{-2}$	16:0, 18:1, 16:1, 14:0, 19:1, 14:1	8.6	25.4 ± 0.6	0.6	9.6×10^{-5}
CL 68:3	4.86	700.486	$[M-2H]^{-2}$	18:1, 16:0, 16:1	-0.7	81.4 ± 2.4	0.3	1.5×10^{-5}
CL 69:4	4.81	706.491	$[M-2H]^{-2}$		5.7	5.5 ± 0.1	0.5	7.5×10^{-5}
CL 69:3	4.86	707.492	$[M-2H]^{-2}$	16:0, 18:1, 16:1, 19:1	-3.9	34.2 ± 0.9	0.5	9.1×10^{-5}
CL 70:3	4.89	714.500	$[M-2H]^{-2}$	18:1, 16:0, 16:1, 19:1	-2.8	32.7 ± 0.6	0.4	1.3×10^{-5}
CL 71:3	4.96	721.503	$[M-2H]^{-2}$		-9.5	9.6 ± 0.2	0.5	6.2×10^{-5}
CL 72:4	4.86	727.508	$[M-2H]^{-2}$		-3.2	5.1 ± 0.1	0.4	3.4×10^{-5}
LysylPG 30:1	7.72	821.565	$[M+H]^{+}$		-0.6	5.1 ± 0.7	0.3	0.0022
LysylPG 30:0	7.72	823.580	$[M+H]^{+}$		-1.3	4.4 ± 0.8	0.4	0.0086
LysylPG 32:2	7.72	847.582	$[M+H]^{+}$	16:1, 18:1	1.2	4 ± 0.6	0.3	0.0024
LysylPG 32:1	7.72	849.597	$[M+H]^{+}$	14:0, 16:0, 18:1. 16:1	0.3	26.7 ± 4.6	0.3	0.0043
LysylPG 34:2	7.68	875.614	$[M+H]^{+}$	16:1, 18:1	2.6	11.3 ± 1.7	0.3	0.0026
LysylPG 34:1	7.68	877.628	$[M+H]^{+}$	16:0, 18:1	0.5	60.2 ± 10.3	0.2	0.0033
LysylPG 35:2	7.65	889.627	$[M+H]^{+}$		-0.6	5.7 ± 0.8	0.4	0.0024
LysylPG 35:1	7.68	891.642	$[M+H]^{+}$	16:0, 19:1	-1.5	17.9 ± 3	0.5	0.011
LysylPG 36:2	7.65	903.643	$[M+H]^{+}$	18:1	-0.7	11.5 ± 1.8	0.3	0.0030
