# Supplementary material for: Characterization of the Mechanisms of Daptomycin Resistance among Gram-Positive Bacterial Pathogens by Multidimensional Lipidomics
Source: mSphere. 2017 Dec 13;2(6):e00492-17. doi: 10.1128/mSphere.00492-17 (PMC5729219; doi:10.1128/mSphere.00492-17)
Supplement: TABLE S4 [file sph006172426st4.pdf]

Putative ID	Ret. Time (min)	*m/z*	Adduct	Fatty Acids [a]	Accuracy (ppm)	N315 ($x10^3$)	Fold (N315-D8/N315)	*P*-value [b]
FA 14:0	0.77	227.200	$[M-H]^-$		-6.9	9.1 ± 0.3	0.4	2.5×10^{-5}
FA 15:0	0.77	241.218	$[M-H]^-$		1.7	24.3 ± 0.7	1.2	0.0067
FA 16:0	0.77	255.234	$[M-H]^-$		3.2	71.0 ± 0.7	0.8	1.8×10^{-5}
FA 17:0	0.74	269.248	$[M-H]^-$		-3.8	14.1 ± 0.4	1.8	6.9×10^{-5}
FA 18:1	0.71	281.248	$[M-H]^-$		-0.7	34.8 ± 0.9	0.6	5.7×10^{-5}
FA 18:0	0.74	283.266	$[M-H]^-$		4.9	103.5 ± 1.2	0.8	5.1×10^{-5}
FA 19:0	0.74	297.282	$[M-H]^-$		7.5	11.5 ± 0.2	10.4	6.3×10^{-7}
FA 20:0	0.71	311.299	$[M-H]^-$		10.3	46.0 ± 1.0	1.6	3.9×10^{-5}
FA 21:0	0.71	325.315	$[M-H]^-$		10.2	2.1 ± 0.1	20.2	2.0×10^{-8}
FA 22:0	0.67	339.326	$[M-H]^-$		-3.5	4.0 ± 0.2	3.8	2.2×10^{-7}
FA 23:0	0.67	353.341	$[M-H]^-$		-4.3	1.2 ± 0.0	7.8	5.9×10^{-9}
MGDG 31:0	0.59	734.576	$[M+NH_4]^+$		-1.7	9.5 ± 0.3	0.8	0.0017
MGDG 32:0	0.59	748.594	$[M+NH_4]^+$		0.5	10.4 ± 0.3	1.7	2.3×10^{-5}
MGDG 33:0	0.59	762.610	$[M+NH_4]^+$		0.9	21.6 ± 0.9	0.4	3.5×10^{-5}
MGDG 34:0	0.59	776.624	$[M+NH_4]^+$		-0.7	5.7 ± 0.2	3.6	1.7×10^{-7}
MGDG 35:0	0.59	790.640	$[M+NH_4]^+$		-0.2	12.0 ± 0.3	1.3	8.8×10^{-5}
MGDG 36:0	0.59	804.652	$[M+NH_4]^+$		-4.4	2.8 ± 0.1	3.4	4.2×10^{-7}
DGDG 28:0	1.17	854.583	$[M+NH_4]^+$		-0.3	10.0 ± 0.2	0.9	0.0032
DGDG 29:0	1.14	868.599	$[M+NH_4]^+$		-0.5	40.8 ± 0.3	0.5	1.6×10^{-7}
DGDG 30:0	1.14	882.616	$[M+NH_4]^+$	15:0	1.6	122.3 ± 1.2	1.1	3.8×10^{-4}
DGDG 31:0	1.14	896.633	$[M+NH_4]^+$	16:0, 15:0, 17:0, 14:0	2.2	225.2 ± 2.0	0.3	4.6×10^{-8}
DGDG 32:0	1.08	910.649	$[M+NH_4]^+$	17:0, 15:0	3.0	264.1 ± 0.8	1.7	2.5×10^{-9}
DGDG 33:0	1.08	924.665	$[M+NH_4]^+$	18:0, 15:0	3.8	900.6 ± 3.1	0.5	5.8×10^{-9}
DGDG 34:0	1.05	938.680	$[M+NH_4]^+$	19:0, 15:0	2.2	166.0 ± 1.5	2.8	1.9×10^{-9}
DGDG 35:0	1.02	952.697	$[M+NH_4]^+$	20:0, 15:0	3.7	551.9 ± 6.0	1.1	7.6×10^{-5}
DGDG 36:0	0.98	966.708	$[M+NH_4]^+$	21:0, 15:0, 17:0, 19:0	-0.5	44.3 ± 1.0	6.1	6.6×10^{-9}
DGDG 37:0	1.02	980.723	$[M+NH_4]^+$		-1.0	10.5 ± 0.2	2.8	3.9×10^{-8}
DGDG 38:0	1.02	994.735	$[M+NH_4]^+$		-5.1	2.4 ± 0.0	4.4	1.2×10^{-7}
PG p29:1	1.39	661.448	$[M-H]^-$		4.0	93.6 ± 11.5	0.1	4.1×10^{-4}
PG p30:1	1.36	675.463	$[M-H]^-$		3.3	68.2 ± 8.3	0.02	3.4×10^{-4}
PG p31:1	1.36	689.478	$[M-H]^-$		2.8	69.8 ± 8.3	0.2	7.6×10^{-4}
PG p32:1	1.33	703.495	$[M-H]^-$		3.7	112.4 ± 12.7	0.1	3.1×10^{-4}
PG p33:1	1.33	717.508	$[M-H]^-$		-0.1	33.5 ± 4.4	0.4	0.0033
PG p34:1	1.29	731.523	$[M-H]^-$		0.1	60.7 ± 6.8	0.2	4.7×10^{-4}
PG 24:0	2.45	609.378	$[M-H]^-$		1.1	27.7 ± 3.7	0.01	5.0×10^{-4}
PG 25:0	2.41	623.396	$[M-H]^-$		4.5	76.6 ± 8.6	0.1	3.6×10^{-4}
PG 26:0	2.38	637.408	$[M-H]^-$		-0.8	16.6 ± 2.1	0.1	4.7×10^{-4}
PG 27:0	2.32	651.424	$[M-H]^-$		-0.6	19.1 ± 2.2	0.2	5.3×10^{-4}
PG 28:0	2.29	665.441	$[M-H]^-$		1.2	57.9 ± 5.5	0.1	1.6×10^{-4}
PG 29:0	2.26	679.457	$[M-H]^-$		2.2	140.7 ± 11.4	0.04	7.1×10^{-5}

PG 30:0	2.22	693.475	$[M-H]^-$	15:0, 14:0, 16:0	5.9	503.0 ± 38.3	0.1	8.2×10^{-5}
PG 31:0	2.17	707.490	$[M-H]^-$	15:0, 16:0	4.5	598.9 ± 48.9	0.0	7.8×10^{-5}
PG 32:0	2.17	721.506	$[M-H]^-$	15:0, 17:0, 14:0, 18:0	4.7	519.9 ± 39.2	0.3	2.6×10^{-4}
PG 33:0	2.10	735.521	$[M-H]^-$	15:0, 18:0	4.4	807.9 ± 59.2	0.1	6.2×10^{-5}
PG 34:0	2.01	749.535	$[M-H]^-$	15:0, 19:0, 14:0, 20:0	1.8	226.6 ± 15.7	0.8	0.011
PG 35:0	2.01	763.552	$[M-H]^-$	15:0, 20:0	3.8	413.5 ± 23.9	0.3	5.6×10^{-5}
PG 36:0	1.98	777.563	$[M-H]^-$	15:0, 21:0	-2.2	15.9 ± 1.1	3.6	2.3×10^{-5}
CL 55:0	4.81	612.406	$[M-2H]^{-2}$		-4.0	1.4 ± 0.2	0.001	2.5×10^{-4}
CL 56:0	4.81	619.414	$[M-2H]^{-2}$	15:0, 18:0, 16:0, 14:0, 17:0	-4.0	2.1 ± 0.2	0.0001	2.5×10^{-4}
CL 57:0	4.86	626.421	$[M-2H]^{-2}$	15:0, 14:0, 18:0, 17:0, 16:0	-4.6	1.6 ± 0.2	0.02	1.5×10^{-4}
CL 58:0	4.86	633.430	$[M-2H]^{-2}$	15:0, 18:0, 14:0, 20:0, 13:0	-3.7	2.3 ± 0.2	0.005	4.9×10^{-5}
CL 59:0	4.89	640.436	$[M-2H]^{-2}$		-6.1	1.2 ± 0.1	0.1	6.4×10^{-5}
CL 60:0	4.89	647.445	$[M-2H]^{-2}$	15:0, 14:0. 16:0, 18:0	-4.0	1.9 ± 0.1	0.03	4.5×10^{-5}
CL 61:0	4.93	654.453	$[M-2H]^{-2}$	15:0, 16:0, 14:0	-3.6	1.7 ± 0.0	0.03	8.6×10^{-7}
CL 62:0	5.01	661.461	$[M-2H]^{-2}$	15:0, 16:0, 18:0, 14:0, 17:0, 13:0	-3.7	2.1 ± 0.1	0.1	1.4×10^{-5}
CL 63:0	5.05	668.468	$[M-2H]^{-2}$	15:0, 14:0, 18:0	-3.9	2.7 ± 0.2	0.0	1.7×10^{-5}
PA 29:0	6.20	605.416	$[M-H]^-$		-4.7	2.4 ± 0.1	0.3	6.5×10^{-5}
PA 30:0	6.41	619.434	$[M-H]^-$	15:0	-1.4	14.1 ± 0.6	0.9	0.27
PA 31:0	6.17	633.448	$[M-H]^-$	16:0, 15:0	-1.9	12.3 ± 2.1	0.3	0.0040
PA 32:0	6.44	647.463	$[M-H]^-$	17:0, 15:0	-3.8	20.4 ± 1.5	2.6	3.9×10^{-4}
PA 33:0	6.36	661.479	$[M-H]^-$	18:0, 15:0	-3.3	17.7 ± 4.4	0.5	0.069
PA 34:0	6.41	675.494	$[M-H]^-$	19:0, 15:0	-4.2	12.4 ± 1.5	3.2	0.0082
PA 35:0	6.36	689.509	$[M-H]^-$	20:0, 15:0	-5.7	6.4 ± 1.9	1.9	0.085
PA 36:0	6.41	703.525	$[M-H]^-$	21:0, 15:0	-4.8	1.2 ± 0.2	7.3	0.020
LysylPG 24:0	7.91	739.488	$[M+H]^+$		1.8	16.7 ± 0.3	0.1	1.3×10^{-7}
LysylPG 25:0	7.87	753.505	$[M+H]^+$		2.8	50.4 ± 0.6	0.2	1.0×10^{-7}
LysylPG 26:0	7.84	767.519	$[M+H]^+$		0.9	7.3 ± 0.1	0.2	3.4×10^{-7}
LysylPG 27:0	7.84	781.534	$[M+H]^+$		0.6	8.8 ± 0.3	0.3	5.7×10^{-6}
LysylPG 28:0	7.80	795.550	$[M+H]^+$		1.2	29.8 ± 0.8	0.1	9.6×10^{-7}
LysylPG 29:0	7.75	809.567	$[M+H]^+$		2.2	80.2 ± 2.0	0.04	6.5×10^{-7}
LysylPG 30:0	7.72	823.586	$[M+H]^+$	15:0	6.2	318.1 ± 6.1	0.1	3.9×10^{-7}
LysylPG 31:0	7.68	837.601	$[M+H]^+$	15:0, 16:0	5.0	328.6 ± 5.3	0.1	1.7×10^{-7}
LysylPG 32:0	7.68	851.615	$[M+H]^+$	15:0, 17:0	4.1	266.3 ± 5.3	0.5	5.6×10^{-6}
LysylPG 33:0	7.65	865.631	$[M+H]^+$	15:0, 18:0	4.1	420.9 ± 5.2	0.1	2.4×10^{-7}
LysylPG 34:0	7.65	879.644	$[M+H]^+$	15:0, 19:0	0.2	92.8 ± 2.5	1.2	4.0×10^{-4}
LysylPG 35:0	7.60	893.661	$[M+H]^+$	15:0, 20:0	1.8	164.1 ± 2.4	0.3	4.8×10^{-7}
LysylPG 36:0	7.60	907.669	$[M+H]^+$		-5.9	6.6 ± 0.4	3.1	2.3×10^{-6}
