# Supplementary figures and images for: Characterization of the Mechanisms of Daptomycin Resistance among Gram-Positive Bacterial Pathogens by Multidimensional Lipidomics
Source: mSphere. 2017 Dec 13;2(6):e00492-17. doi: 10.1128/mSphere.00492-17 (PMC5729219; doi:10.1128/mSphere.00492-17)

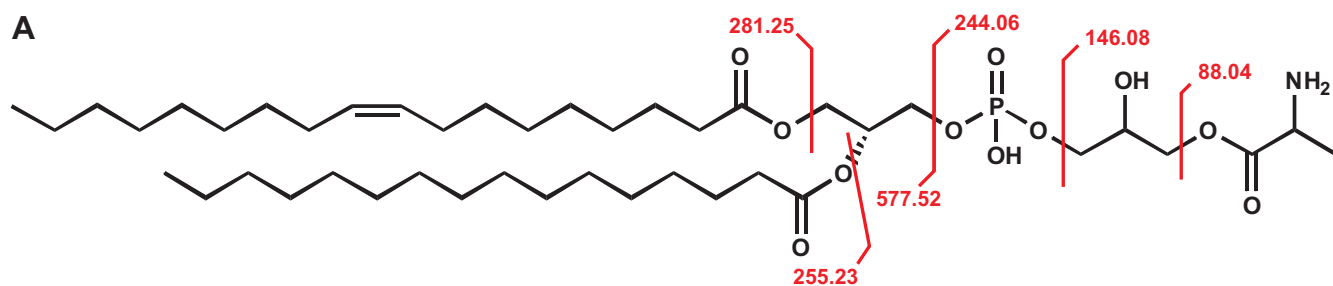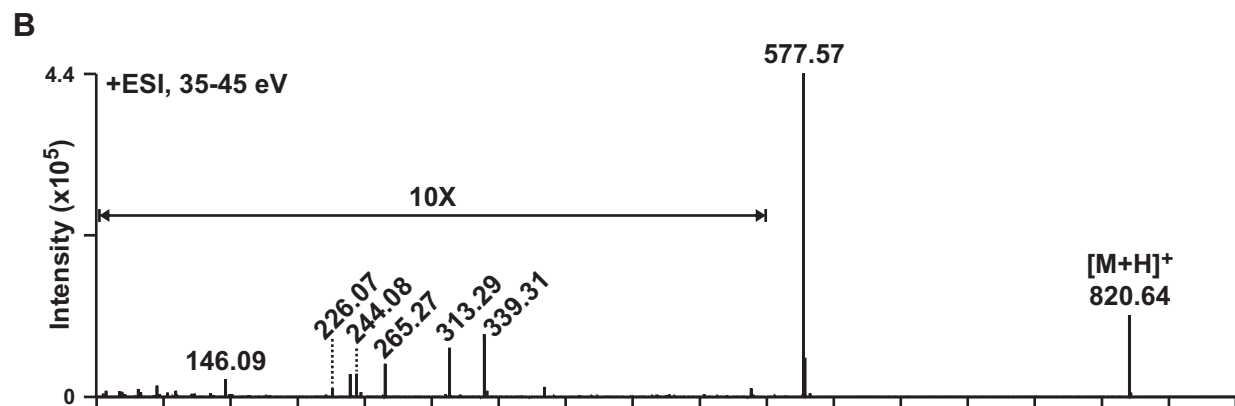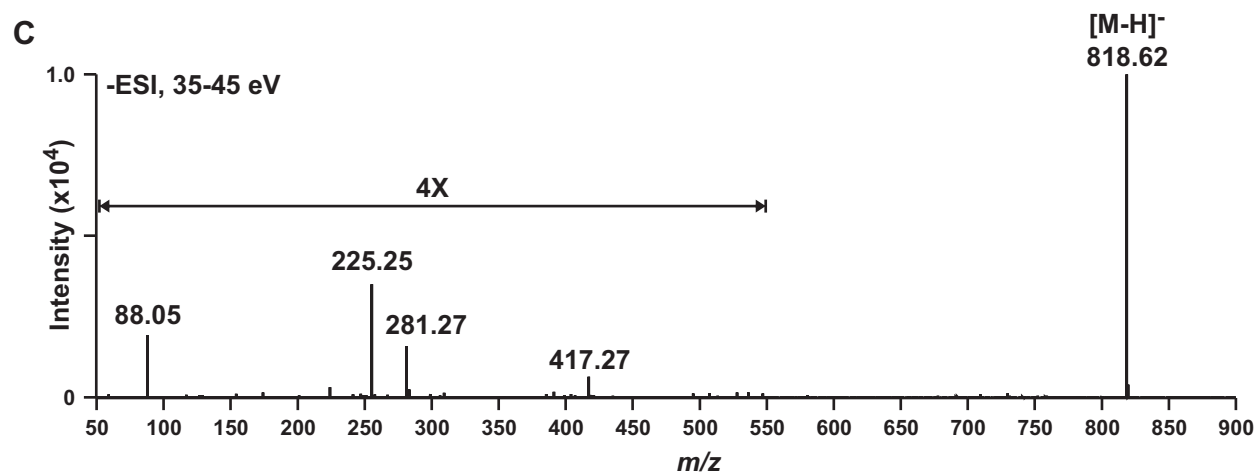

Supplement: FIG S1 [file sph006172426sf6.pdf]

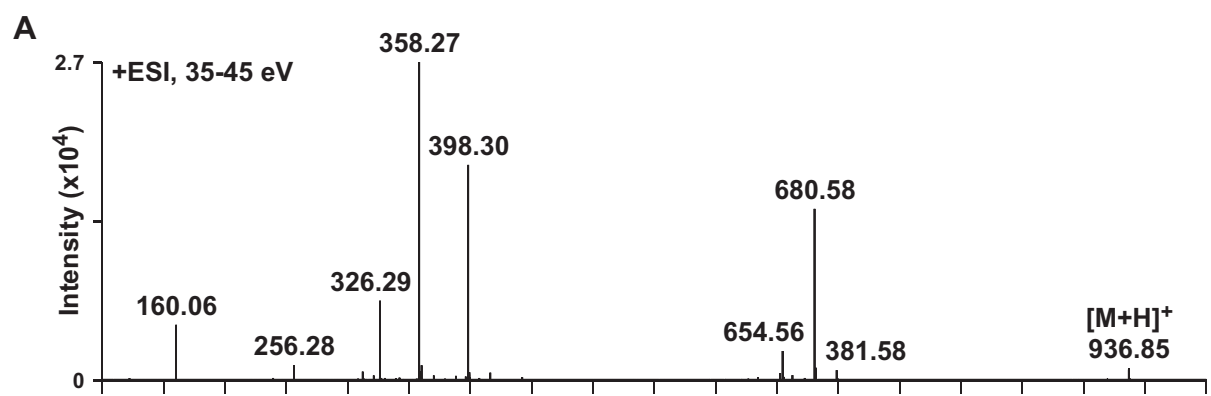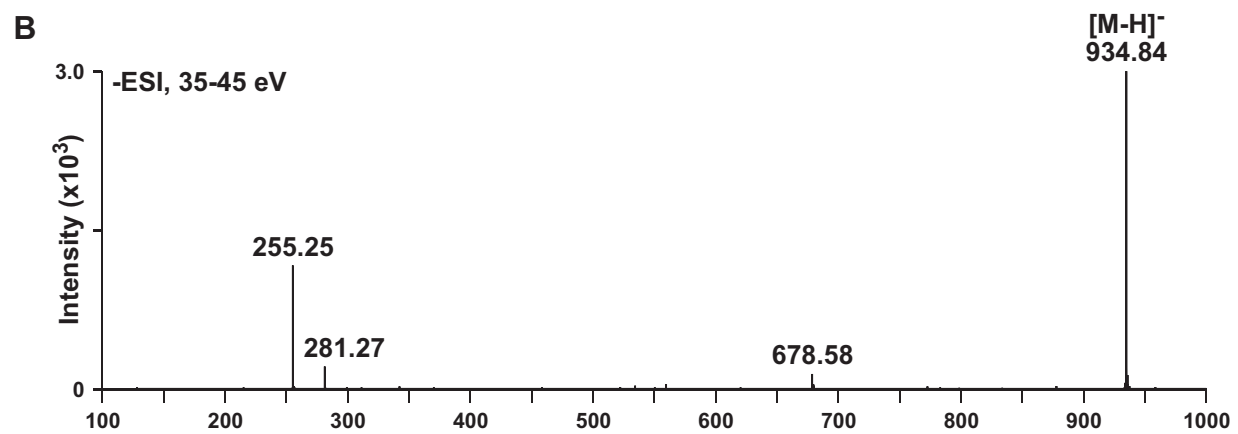

Supplement: FIG S2 [file sph006172426sf7.pdf]

**A**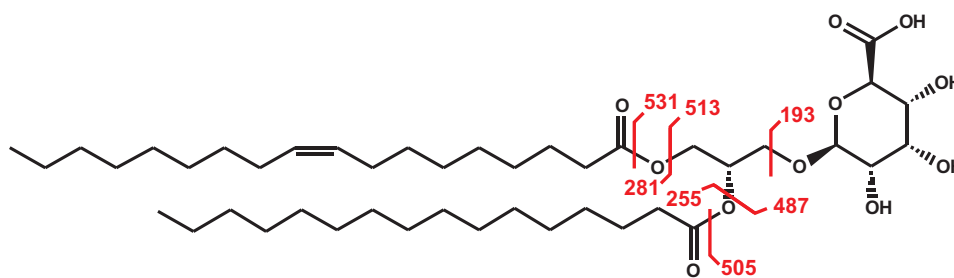**B**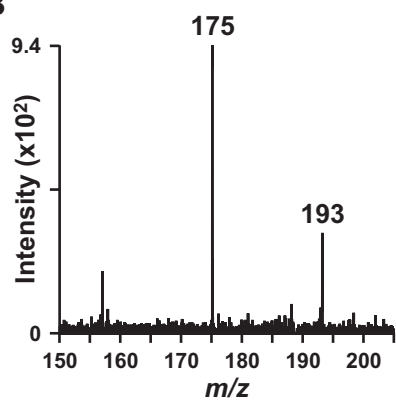**C**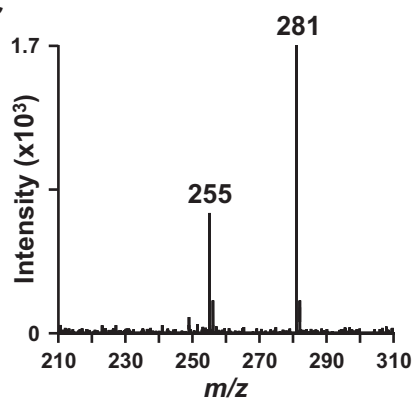**D**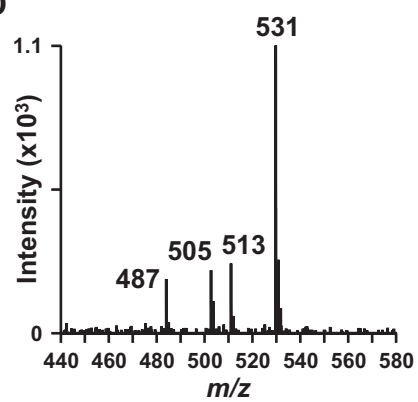

Supplement: FIG S3 [file sph006172426sf8.pdf]

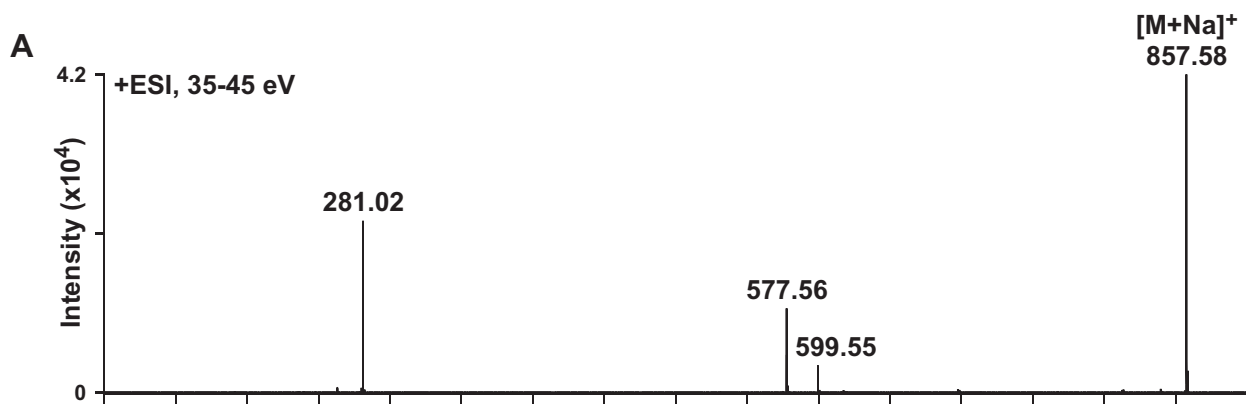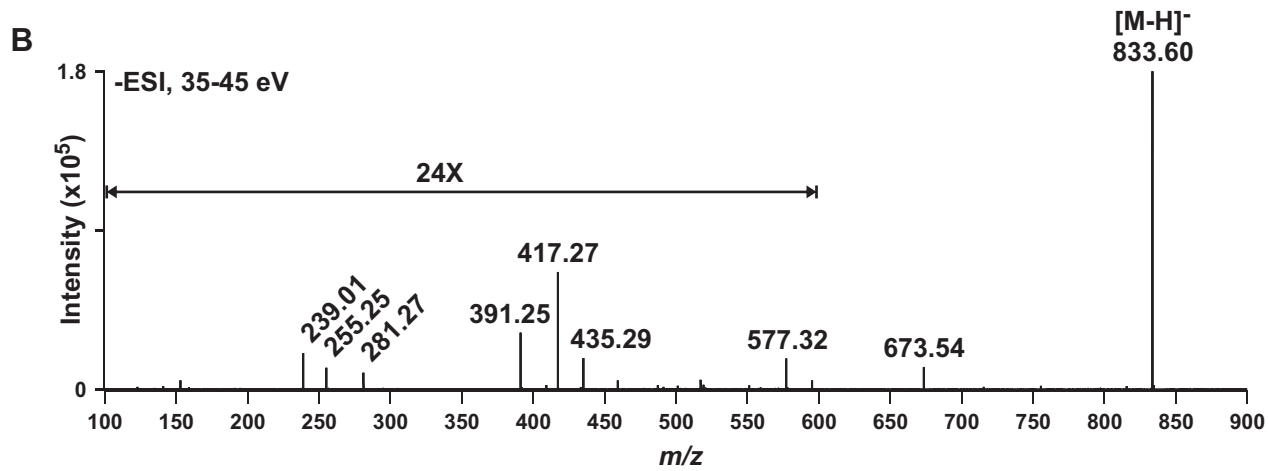

Supplement: FIG S4 [file sph006172426sf9.pdf]
