# Supplementary material for: Characterization of the Mechanisms of Daptomycin Resistance among Gram-Positive Bacterial Pathogens by Multidimensional Lipidomics
Source: mSphere. 2017 Dec 13;2(6):e00492-17. doi: 10.1128/mSphere.00492-17 (PMC5729219; doi:10.1128/mSphere.00492-17)
Supplement: TABLE S5 [file sph006172426st5.pdf]

| **Putative ID** | **Ret. Time (min)** | ***m/z*** | **Adduct** | **Fatty Acids** [a] | **Accuracy (ppm)** | **W40308 ($x10^3$)** | **Fold (W49297/ W40308)** | ***P*-value** [b] |
|---|---|---|---|---|---|---|---|---|
| FA 18:1 | 0.71 | 281.248 | $[M-H]^-$ | | -2.2 | 30.2 ± 2.4 | 2.0 | $5.0 \times 10^{-5}$ |
| FA 16:0 | 0.74 | 255.235 | $[M-H]^-$ | | 7.6 | 136.3 ± 1.5 | 1.1 | $1.7 \times 10^{-4}$ |
| 0.90_934.7546m/z | 0.90 | 934.755 | $[M-H]^-$ | 16:0, 18:1 | | 43.9 ± 1.0 | 0.2 | $1.4 \times 10^{-6}$ |
| 1.36_857.5184m/z | 1.36 | 857.518 | $[M+Na]^+$ | 18:1, 16:0 | | 181.2 ± 29.8 | 3.9 | $2.8 \times 10^{-5}$ |
| PG 16:0-18:1 | 2.01 | 747.523 | $[M-H]^-$ | 16:0, 18:1 | 5.8 | 1050.4 ± 34.6 | 0.04 | $2.1 \times 10^{-6}$ |
| PG 32:0 | 2.10 | 721.505 | $[M-H]^-$ | 16:0 | 3.8 | 387.1 ± 17.9 | 0.03 | $7.7 \times 10^{-6}$ |
| GlcADG 16:0-18:1 | 2.22 | 788.591 | $[M+NH_4]^+$ | 16:0, 18:1 | 3.9 | 208.4 ± 7.8 | 3.3 | $3.4 \times 10^{-5}$ |
| GlcADG 16:0-16:0 | 2.29 | 767.524 | $[M+Na]^+$ | 18:1, 16:0 | -4.8 | 103.0 ± 8.9 | 2.6 | $4.0 \times 10^{-5}$ |
| PI 16:0-18:1 | 3.57 | 859.536 | $[M+Na]^+$ | 18:1, 16:0 | 5.7 | 487.9 ± 134.3 | 2.7 | 0.0011 |
| PI 16:0-16:0 | 3.72 | 833.517 | $[M+Na]^+$ | 16:0 | 2.4 | 103.0 ± 33.4 | 1.9 | 0.018 |
| AlaPG 16:0-18:1 | 4.46 | 820.574 | $[M+H]^+$ | 16:0, 18:1 | 4.8 | 305.9 ± 65.8 | 0.1 | 0.0049 |
| CL 68:2 | 4.89 | 701.497 | $[M-2H]^{-2}$ | 16:0, 18:1 | 3.5 | 232.7 ± 51.5 | 0.05 | 0.0037 |
| CL 66:2 | 4.86 | 687.478 | $[M-2H]^{-2}$ | 16:0, 18:1, 14:0, 16:1 | -0.6 | 81.0 ± 27.2 | 0.03 | 0.015 |
| PA 16:0-18:1 | 6.32 | 673.482 | $[M-H]^-$ | 18:1, 16:0 | 0.9 | 35.2 ± 6.9 | 2.8 | $2.2 \times 10^{-4}$ |
| PA 16:0-16:0 | 6.32 | 647.466 | $[M-H]^-$ | 16:0 | 0.9 | 46.7 ± 2.0 | 4.4 | $4.0 \times 10^{-8}$ |
